# Structure Analysis and Conformational Transitions of the Cell Penetrating Peptide Transportan 10 in the Membrane-Bound State

**DOI:** 10.1371/journal.pone.0099653

**Published:** 2014-06-17

**Authors:** Susanne Fanghänel, Parvesh Wadhwani, Erik Strandberg, Wouter P. R. Verdurmen, Jochen Bürck, Sebastian Ehni, Pavel K. Mykhailiuk, Sergii Afonin, Dagmar Gerthsen, Igor V. Komarov, Roland Brock, Anne S. Ulrich

**Affiliations:** 1 Karlsruhe Institute of Technology (KIT), Institute of Organic Chemistry and DFG-Center for Functional Nanostructures (CFN), Karlsruhe, Germany; 2 KIT, Institute of Biological Interfaces (IBG2), Karlsruhe, Germany; 3 Department of Biochemistry, Radboud Institute for Molecular Life Sciences, Radboud University Medical Centre, Nijmegen, The Netherlands; 4 Taras Shevchenko National University of Kyiv, Chemistry Department, Kyiv, Ukraine and Enamine Ltd., Kyiv, Ukraine; 5 KIT, Laboratory for Electron Microscopy, Karlsruhe, Germany; 6 Taras Shevchenko National University of Kyiv, Institute of High Technologies, Kyiv, Ukraine; University of Pittsburgh School of Medicine, United States of America

## Abstract

Structure analysis of the cell-penetrating peptide transportan 10 (TP10) revealed an exemplary range of different conformations in the membrane-bound state. The bipartite peptide (derived N-terminally from galanin and C-terminally from mastoparan) was found to exhibit prominent characteristics of (i) amphiphilic α-helices, (ii) intrinsically disordered peptides, as well as (iii) β-pleated amyloid fibrils, and these conformational states become interconverted as a function of concentration. We used a complementary approach of solid-state ^19^F-NMR and circular dichroism in oriented membrane samples to characterize the structural and dynamical behaviour of TP10 in its monomeric and aggregated forms. Nine different positions in the peptide were selectively substituted with either the ***L*** **-** or ***D*** **-**enantiomer of 3-(trifluoromethyl)-bicyclopent-[1.1.1]-1-ylglycine (CF_3_ *-*Bpg) as a reporter group for ^19^F-NMR. Using the ***L*** **-**epimeric analogs, a comprehensive three-dimensional structure analysis was carried out in lipid bilayers at low peptide concentration, where TP10 is monomeric. While the N-terminal region is flexible and intrinsically unstructured within the plane of the lipid bilayer, the C-terminal α-helix is embedded in the membrane with an oblique tilt angle of ∼55° and in accordance with its amphiphilic profile. Incorporation of the sterically obstructive ***D*** **-**CF_3_ *-*Bpg reporter group into the helical region leads to a local unfolding of the membrane-bound peptide. At high concentration, these helix-destabilizing C-terminal substitutions promote aggregation into immobile β-sheets, which resemble amyloid fibrils. On the other hand, the obstructive ***D*** **-**CF_3_ *-*Bpg substitutions can be accommodated in the flexible N-terminus of TP10 where they do not promote aggregation at high concentration. The cross-talk between the two regions of TP10 thus exerts a delicate balance on its conformational switch, as the presence of the α-helix counteracts the tendency of the unfolded N-terminus to self-assemble into β-pleated fibrils.

## Introduction

Membrane-active peptides are often used as cell-penetrating carriers and antimicrobial agents, but their actual structural behavior in the membrane-bound state remains elusive and makes rational design difficult. Interactions with the lipid bilayer tend to induce specific peptide conformations or trigger structural changes, such as folding, oligomerization, or aggregation into amyloid-like fibrils. Here, we have characterized a representative cell-penetrating peptide, transportan 10 (TP10), and observed a remarkable structural complexity and conformational equilibrium. The detailed insights gained into the stabilities and interconversions of its α-helical, unfolded and β-pleated states reveal some general principles on peptide folding and aggregation in membranes.

Cell-penetrating peptides (CPPs) are used to deliver hydrophilic cargo into cells without disrupting the plasma membrane [1]–[6]. Different classes of CPPs have been described, such as the arginine-rich transporters represented by oligo-arginine and the well-known TAT peptide [7], [8] on the one hand, or the cationic amphiphilic peptides exemplified by penetratin [2], [6], [9]–[11] and the transportan family [12]–[16] on the other hand. CPPs differ in their mechanism of uptake, as transportan peptides tend to be more membrane-perturbing than arginine-rich ones [17], [18]. In fact, transportan shares many characteristics with pore-forming α-helical antimicrobial peptides and toxins, such as magainin 2 and melittin [19]–[22]. Given their structural similarities, some CPPs have been shown to act like antimicrobial peptides, and *vice versa* [19], [23]–[27]. Another notable feature of many man-made peptides is their tendency to aggregate at high concentration [28], [29], a characteristic shared with natural fibril-forming peptides such as the toxic Aβ peptide [30]–[34], α-synuclein [35], [36], IAPP [37], or calcitonin [38]. In many of these systems, aggregation is surface-induced or at least enhanced upon binding to lipid membranes [28]–[30], [32], [35]–[37], [39], [40].

Peptides are often classified as cell penetrating, membrane permeabilizing, lytic or fusogenic, and all of them induce some kind of perturbation in the lipid bilayer. It is generally not possible to correlate either of these events with any particular peptide structure, as many peptides (i) are multifunctional [23], [25]–[27], [41], (ii) have non-trivial membrane-bound conformations [42], and (iii) can interconvert between several different structures [43]. Here, we set out to understand the diverse ways how a single representative peptide can interact with membranes. Such a comprehensive view on the conformational transitions and aggregation behavior should then be highly valuable for the wider area of membrane-active peptides in general.

We focused on the membrane-bound structures and the conformational transitions of TP10, a shorter analog of the original transportan (TP) peptide with reduced toxicity [4], [16], [21], [22]. This chimeric family consist N-terminally of a sequence derived from the neuropeptide galanin [44], and C-terminally of a sequence from the wasp venom, mastoparan [16], [45]. The two parts are linked by an extra Lys residue, and the hybrid peptides are highly cationic. They have been successfully used to transport a variety of biologically relevant cargoes into living cells [4], [46], though the detailed steps of internalization are still controversial. The structure of TP in a membrane-mimetic environment has been resolved using liquid-state NMR in SDS micelles and in phospholipid bicelles [12], [47]. A well-defined α-helix is reported for the C-terminal mastoparan part, while the N-terminal galanin region is more disordered. The hinge between the two segments is located around Asn15 (equivalent to Asn9 in TP10). However, this picture neither reveals the interaction with nor the alignment of the peptide in a planar lipid membrane. Furthermore, TP has a tendency to aggregate, like many other cell-penetrating peptides in the membrane-bound state [28], [29]. To understand and control the membrane interactions of this representative cell-penetrating peptide, an analysis of its detailed conformation and conformational transitions in contact with the lipid bilayer is required. Such insight is a prerequisite for optimizing any peptide sequences that are associated with cell uptake or applied to disrupt membranes.

Our strategy for investigating the membrane-bound peptide is based on two complementary techniques, namely solid-state NMR and oriented circular dichroism (OCD) [48]–[50]. Both methods make use of macroscopically aligned lipid bilayer samples, in which the peptide can be studied under quasi-native conditions, i.e., at ambient temperature, adequate hydration, and with a well-defined lipid composition and peptide-to-lipid ratio. OCD provides rapid qualitative information about the conformation and alignment of the peptide [48], while solid-state NMR can yield a full structure with quasi-atomic resolution [39], [40], [49], [51]–[56]. Especially ^19^F-NMR analysis of selectively ^19^F-labeled peptides is a highly sensitive approach to obtain site-specific information [39], [40], [49], [51]–[56], similar to an alanine or cysteine scan used in molecular genetics. By introducing a single CF_3_-labeled amino acid into successive positions along the peptide backbone, a three-dimensional picture of the molecule in the lipid bilayer can be obtained. Further information on local and global peptide dynamics can be extracted from the effects of motional averaging [39], [40], [49], [51]–[57]. For several peptides it has already been possible to describe various concentration-dependent effects, such as the re-alignment of α-helices [51], [58]–[60] or the aggregation into β-sheets [39], [40], from which mechanistic insights could be deduced. With a typical length of 10 to 30 amino acids, all peptides studied so far have exhibited just one type of secondary structure in any particular membrane-bound state. Interestingly, we find here that the 21-mer TP10 possesses a distinct bipartite structure, in which the N- and C-terminal regions adopt different conformations, and perturbations in these two regions elicit a differential sensitivity towards aggregation.

The solid-state ^19^F-NMR approach relies on the designer-made ^19^F-labeled amino acid 3-(trifluoromethyl)-bicyclopent-[1.1.1]-1-ylglycine (CF_3_-Bpg), which has a stiff and sterically restrictive side chain [52], [54], [61], [62]. The ***L*** **-**enantiomer has been recently established as a selective NMR label in studies of membrane-bound peptides with simple α-helical or β-pleated conformations [40], [52], [54], [63], [64]. By incorporating ***D*** **-**amino acids into peptides, it has furthermore been possible to modulate their ability to aggregate as β-sheets [39], [65], [66]. Now that ^19^F-NMR has been established as a reliable method, it is possible to address the bipartite structure and complex conformational transitions of the functionally interesting peptide TP10 in detail. This example shows for the first time how a hybrid peptide is embedded in a membrane with two different conformational elements, and how a local destabilization in the α-helical region promotes extensive aggregation into β-sheeted amyloid-like fibrils. By incorporating the sterically constrained CF_3_-Bpg into specific regions of TP10, we could thus observe and control its conformational transitions.

## Materials and Methods

### Materials

All amino acids were purchased from Novabiochem (Läufelfingen, Switzerland) or IRIS Biotech (Marktredwitz, Germany), and the coupling reagents 2-(1H-benzotriazol-1-yl)-1,1,3,3-tetramethyluroniumhexafluorophosphate (HBTU) and 1-hydroxybenzo-triazol (HOBt) from Biosolve (Valkenswaard, Netherlands). The ^19^F-labeled amino acid CF_3_ *-*Bpg was prepared in the enantiomerically pure ***L*** **-** and ***D*** **-**forms by Enamine Ltd. (Kyiv, Ukraine). The lipids 1,2-dimyristoyl-*sn*-glycero-3-phosphocholine (DMPC) and 1,2-dimyristoyl-*sn*-glycero-3-[phospho-rac-(1-glycerol)] (DMPG) were obtained from Avanti Polar Lipids (Alabaster, AL, USA). RPMI 1640 and fetal calf serum (FCS) were from PAN Biotech (Aidenbach, Germany).

### Solid-phase Peptide Synthesis

All peptides were synthesized on an automated Syro II multiple peptide synthesizer (MultiSynTech, Witten, Germany) with standard Fmoc solid-phase peptide synthesis protocols [67] and HOBt/HBTU as coupling reagents. The ^19^F-labeled amino acid CF_3_-Bpg (see Scheme S1 in File S1) was coupled manually for 2 h as previously described [40]. The peptides were cleaved off the resin using a mixture of trifluoroacetic acid (TFA) (93.5%), triisopropylsilane (TIS) (4%) and H_2_O (2.5%), precipitated with diethyl ether and lyophilized. The crude peptides were purified by high-performance liquid chromatography (HPLC) on a preparative C18 column (22 mm×250 mm) (Vydac, Hesperia, CA, USA) using acetonitrile water gradients supplemented with 5 mM HCl. The identity of all peptides was confirmed by mass spectrometry. The purity of the peptides was found to be over 95%.

### Carboxyfluorescein (CF)-labeling of Peptides

Peptides were N-terminally coupled to CF before being cleaved off the resin. Diisopropylcarbodiimide, HOBt and 5(6)-CF in a molar ratio of 1∶1∶1 were dissolved in dimethylformamide (DMF), mixed with the peptide on the resin in a molar ratio of 5∶1, and coupled for 12 h. After washing with DMF, dichloromethane (DCM), methanol (MeOH) and diethyl ether, piperidine (20% v/v in DMF) was added for 30 min. Afterwards the resin was washed with DMF, DCM and MeOH, dried under reduced pressure, and the peptide was cleaved off the resin and purified using HPLC.

### Cell Uptake Assay with Fluorescence Microscopy

HeLa cells were maintained in RPMI 1640 supplemented with 10% FCS, and incubated at 37°C in a 5% CO_2_-containing humidified incubator. Cells were passaged every 2 to 3 days. The concentrations of the CF-labeled peptides were determined by measuring A_492_ in Tris-HCl buffer (pH 8.8), assuming a molar extinction coefficient of 75,000 M^−1 ^cm^−1^. Confocal laser scanning microscopy was performed on a TCS SP5 confocal microscope (Leica Microsystems, Mannheim, Germany) equipped with an HCX PL APO 63×N.A. 1.2 water immersion lens. HeLa cells were maintained at 37°C on a temperature-controlled microscope stage. The cells were seeded at a density of 10,000 cells/well three days before the experiment in 8-well microscopy chambers (Nunc, Wiesbaden, Germany) and grown to 75% confluence. Cells were incubated with 2 or 10 µM of the CF-labeled TP10 wild type (WT) or the analogs in RPMI 1640 supplemented with 10% FCS for 30 min at 37°C. Cells were washed twice after the incubation, and living cells were analyzed immediately by confocal microscopy.

### Circular Dichroism Spectroscopy

Circular dichroism (CD) experiments were performed on a J-815 spectropolarimeter (Jasco, Tokyo, Japan) over the range from 260 to 180 nm at 0.1 nm intervals, using a quartz glass cuvette of 1 mm optical path length (Suprasil, Hellma Optik, Jena, Germany). The spectra were recorded using a scan rate of 10 nm/min, 8 s response time and 1 nm bandwidth. Three scans were averaged and the baseline spectrum of pure lipids was subtracted. The spectra of the peptides in the presence of lipids were recorded at 30°C (i.e. above the lipid phase transition temperature). The appropriate amounts of peptides were dissolved in phosphate buffer (10 mM, pH 7) to yield a stock solution with a concentration of 0.5 mg/ml. Appropriate amounts of DMPC and DMPG in a molar ratio of 3∶1 were co-dissolved in CHCl_3_/MeOH, dried in a stream of nitrogen followed by drying overnight under reduced pressure, and suspended in 10 mM phosphate buffer, pH 7.0. The lipid dispersion was homogenized by vigorously vortexing for 10×1 min and by 10 freeze-thaw cycles. Afterwards, small unilamellar vesicles (SUVs) were generated by sonication of the multilamellar vesicles for 1 min in a strong ultrasonic bath (UTR 200, Hielscher, Germany). The sonication procedure was repeated 4 times, after cooling the water of the ultrasonic bath down to room temperature with ice, to avoid overheating of the samples. Three different peptide-to-lipid molar ratios (P/L = 1∶50, 1∶100, 1∶200) were tested with a lipid concentration of 1.5 mg/ml and corresponding peptide concentrations of 0.1 mg/ml for P/L of 1∶50, 0.05 mg/ml for a P/L of 1∶100, and 0.025 mg/ml for a P/L of 1∶200.

For secondary structure analysis of TP10, the CD spectrum of the WT peptide at a P/L = 1∶50 in DMPC/DMPG vesicles (see Figure S1 A/B in File S1) was converted to mean residue ellipticities by using the weighed-in peptide amount and the volume of the sample for concentration determination. A reliable UV concentration determination at 280 nm from the absorption of the single Tyr residue contained in the sequence was not possible due to the low absorption values and strong background scattering in the final vesicle sample used for CD. Secondary structure analyses were performed using three different algorithms: the CDSSTR program with the implemented SVD (singular value decomposition) algorithm [68], [69], the CONTIN-LL program, which is based on the ridge regression algorithm [70], [71] and the SELCON-3 program, which incorporates the self-consistent method together with the SVD algorithm to assign protein secondary structure [72], [73]. The three algorithms as well as the used protein CD spectra reference data set #7 are provided by the DICHROWEB on-line server [74], [75]. The quality of the fit between experimental and back-calculated spectrum corresponding to the derived secondary structure fractions was assessed from the normalized root mean square deviation (NRMSD), with a value <0.1 (CONTIN-LL, CDSSTR) and <0.25 (SELCON-3) considered as a good fit [75].

### Oriented Circular Dichroism Spectroscopy (OCD)

OCD experiments on macroscopically oriented samples were performed with a designated OCD sample holder built in-house [48]. Lipids were dissolved in CHCl_3_ and peptides in MeOH, appropriate amounts mixed and spread onto a 12 mm diameter quartz glass plate. The final amount of peptide deposited on a quartz glass plate was 7.5×10^−6 ^mmol, and the amount of lipid was adjusted to obtain desired P/L. After drying under reduced pressure, the samples were hydrated over night at 40°C in 96–97% relative humidity. The spectra were recorded in the range from 260 to 180 nm using a scan rate of 20 nm/min, 8 s response time, 1 nm bandwidth, at eight different rotations of the cell, and referenced by subtracting the background signal that was recorded with a sample containing the same amount of lipids without peptides.

### Solid-state ^19^F-NMR Spectroscopy

All experiments were performed on a Bruker Avance 500 MHz NMR spectrometer (Bruker BioSpin, Rheinstetten, Germany) at 40°C. ^31^P-NMR spectra were acquired at a frequency of 202.5 MHz using a Hahn echo sequence [76] with a 90° pulse of 5 µs, 30 µs echo time, a sweep width of 200 kHz, 4096 data points and 28 kHz proton decoupling with SPINAL64 [77] or TPPM [78]. Usually 128 scans were recorded with a relaxation delay of 1 s. ^19^F-NMR was performed at a frequency of 470.6 MHz using an anti-ringing sequence [79] with a 90° pulse of 3.25 μs, a sweep width of 500 kHz, 4096 data points, and 24 kHz proton decoupling with SPINAL64. Usually, depending on the amount of peptide, between 2,000 (560 µg peptide, P/L = 1∶50) and 20,000 (130 µg peptide, P/L = 1∶400) scans were acquired with a relaxation delay of 1 s.

For preparing the oriented NMR samples, 14–15 mg (8 mg for a P/L of 1∶50) of a DMPC/DMPG mixture in a molar ratio of 3∶1 were dissolved in chloroform and an appropriate amount of peptide depending on the P/L (560 µg for P/L = 1∶50, 260 µg for P/L = 1∶200 and 130 µg for P/L = 1∶400) was dissolved in MeOH. Both solutions were thoroughly mixed and equally distributed on 18 glass plates (15 mm×7.5 mm×0.08 mm) (Marienfeld Laboratory Glassware, Lauda-Königshofen, Germany). The glass plates were stacked and hydrated at 48°C in 96% relative humidity for 24 h after drying overnight under reduced pressure. The hydrated samples were wrapped in parafilm and plastic foil before the NMR experiments.

### NMR Structure Analysis

The orientation of the peptide in the bilayer was calculated based on the experimentally determined dipolar couplings (within the experimental error of 0.5 kHz) of the CF_3_-Bpg labeled TP10 analogs. According to our CD analysis, the backbone was modeled as an ideal α-helix. The alignment of the helix in a bilayer is described by the tilt angle (τ) with respect to the membrane normal, and by the azimuthal rotation angle (ρ) around the helix. The overall effect of motional averaging is taken into account by the Gaussian distribution parameters σ_τ_ and σ_ρ_ as previously described [57]. The orientation of the C_α_−C_β_ bond vector of the ^19^F-labeled side chain is described by the angles α = 121.1° and β = 53.2° [59]. The orientational parameters τ, ρ, σ_τ_ and σ_ρ_ were determined by a least-squares fit. The sum of squared deviations to the experimentally determined dipolar couplings was minimized to find the best-fit parameters.

### Transmission Electron Microscopy (TEM)

The peptides were dissolved in water in a concentration of 2 mM in the absence of lipids. The peptide solution was sprayed on the carbon-coated grids and subsequently stained with uranyl acetate (5% aqueous solution with 30% ethanol) to enhance the contrast. Transmission electron microscopy was performed with a ZEISS 922 microscope at 200 keV electron energy with an in-column Omega filter. The images were taken in the bright-field mode applying zero-loss filtering for contrast enhancement.

## Results

To resolve the structural details and study the aggregation behavior of TP10 in the membrane-bound state, a single ^19^F-NMR reporter group was introduced in the peptide sequence as an ***L*** **-** or ***D*** **-**enantiomer of CF_3_-Bpg at position Gly2, Leu4, Leu5, Ile8, Leu10, Leu13, Leu16, Ile20, or Leu21. All analogs were also synthesized with a CF-label, yielding a total of 36 analogs (*L*-epimers are shown in Table S1 in File S1). The influence of the ^19^F-labeled amino acid on the secondary structure of the peptide was examined by circular dichroism (CD) spectroscopy in solution, and the influence on the biological cell uptake activity was assessed with another set of carboxyfluorescein-^19^F-labeled analogs using fluorescence microscopy on HeLa cells. We then analyzed the ***L*** **-**epimers using solid-state ^19^F-NMR to determine the conformation and orientation of monomeric TP10 at low peptide concentration in oriented DMPC/DMPG (3∶1) bilayers. Thereafter, the ***D*** **-**epimers were measured by ^19^F-NMR and OCD at high peptide concentration to examine their aggregation behavior. The morphology of the TP10 aggregates was characterized by transmission electron microscopy (TEM).

### Characterization of the ^19^F-labeled TP10 Analogs by CD and Cell Uptake Assays

**Figure 1:**
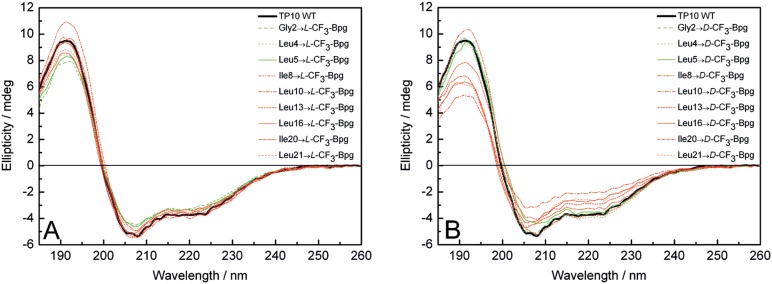
CD spectra of the CF_3_-Bpg labeled TP10 analogs. CD spectra are recorded in the presence of unilamellar DMPC/DMPG (3∶1) vesicles at a P/L ratio of 1∶200. (A) ***L*** **-**epimers and (B) ***D*** **-**epimers are compared with the WT peptide (black line). Analogs with CF_3_-Bpg in the galanin part are represented by green lines and in the mastoparan part by red lines.

To examine the influence of ^19^F-labeling on the secondary structure of TP10, we studied the (WT) peptide and the 18 CF_3_-Bpg labeled analogs by CD spectroscopy in solution. The peptide was added to small unilamellar lipid vesicles composed of DMPC/DMPG (molar ratio of 3∶1) at three different peptide-to-lipid molar ratios (P/L), namely 1∶50, 1∶100 and 1∶200. In these freshly prepared samples, all TP10 analogs have an α-helical conformation similar to the WT peptide at all concentrations tested. CD spectra with a P/L of 1∶200 are shown in Figure 1A and 1B for the ***L*** **-** and ***D*** **-**epimers, respectively. (Data on P/L of 1∶50 and 1∶100 are presented in Figure S1 in File S1). The ***L*** **-**epimers showed no signs of perturbation, neither when the label was incorporated in the N-terminal galanin part, which is colored in green in this and subsequent figures (including residue Ile8, as will become obvious from the data below), nor in the C-terminal mastoparan part (colored in red). We may thus conclude that the TP10 analogs labeled with ***L*** **-**CF_3_-Bpg are essentially unperturbed and can be used for the subsequent ^19^F-NMR structure analysis. Rough secondary structure estimation for the WT peptide by deconvolution of the CD data revealed a helix fraction of around 56%, as shown in Table 1. This corresponds to a stretch of 12 amino acids that are helically folded, while the remaining part of the sequence is predominantly disordered.

**Table 1:** Secondary structure of TP10-WT bound to DMPC/DMPG vesicles evaluated from the CD spectrum (P/L = 1∶50, see Figure S1 A/B in File S1 for details).

Algorithm	Fraction of secondary structure				NRMSD^a^
	α-helix	β-strand	Turn	Unordered	
CONTIN-LL	0.58	0.06	0.12	0.24	0.088
CDSSTR	0.59	0.13	0.12	0.16	0.005
SELCON-3	0.52	0.08	0.17	0.24	0.172

a NRMSD = normalized root mean square deviation between calculated and experimental CD spectra.

The ***D*** **-**epimers showed slight deviations from the WT spectrum, especially when the CF_3_-Bpg label was incorporated in the C-terminal mastoparan region (see red lines in Figure 1B, and in Figures S1B, S1D, and S2B in File S1). It is not surprising that the configuration of ***D*** **-**CF_3_-Bpg leads to moderate structural perturbations due to the inversion of the natural stereochemistry at the backbone.

The additional set of 18 N-terminal carboxyfluorescein-^19^F-labeled TP10 analogs that were prepared for the cell uptake experiments were also characterized by CD, giving essentially the same lineshapes as without the fluorophore (spectra are presented in Figure S2 in File S1). We note already at this point that also the ^19^F-NMR spectra of the ***L*** **-**CF_3_-Bpg labeled peptides with and without carboxyfluorescein showed no significant differences (Figure S3 and Table S2 in File S1), so the fluorescent analogs prepared for the cell-uptake assays can be assumed to represent those used in the structure analysis.

**Figure 2:**
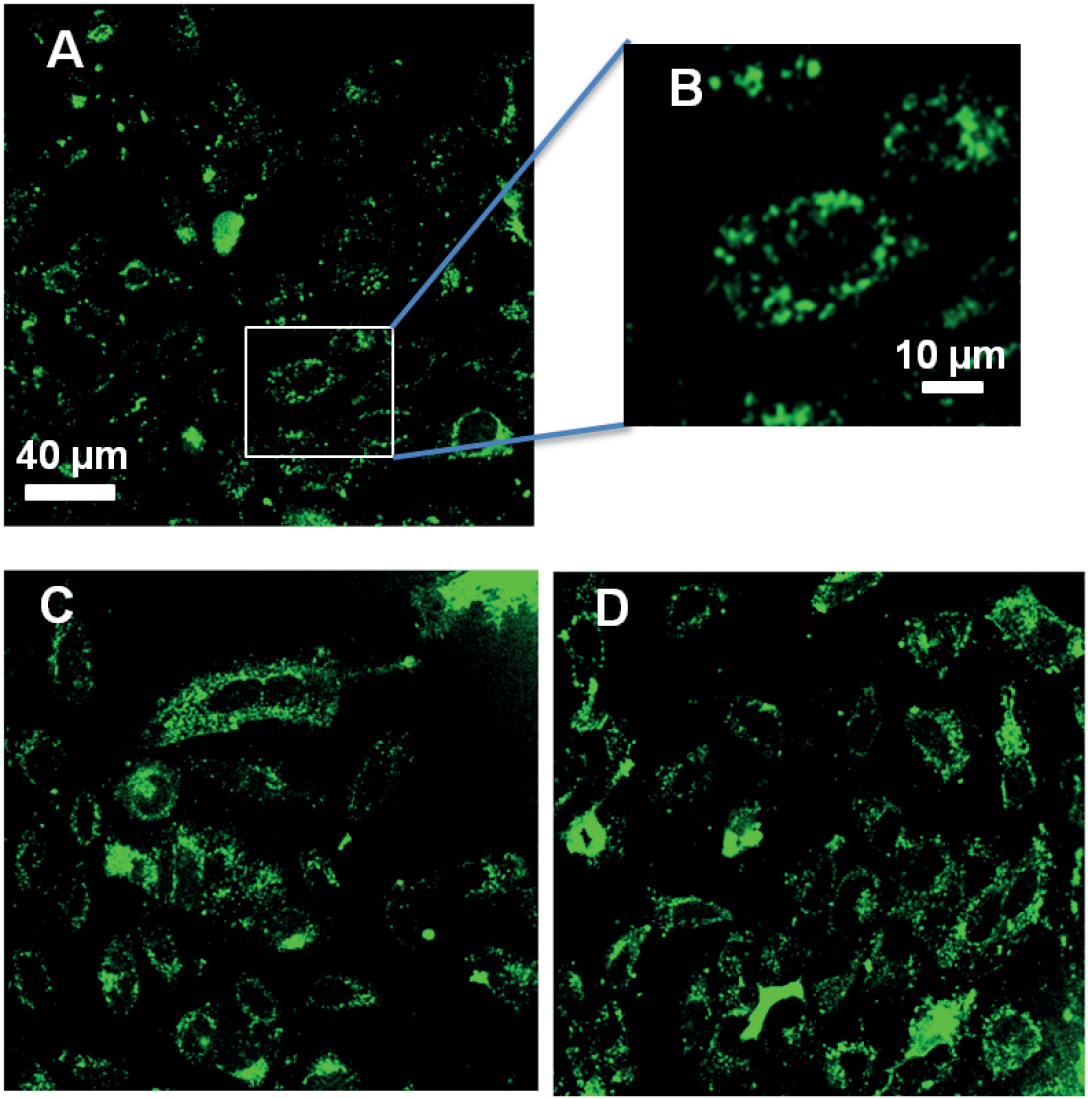
Cellular uptake of TP10. (A, B) Internalization of TP10 WT and of two representative ^19^F-labeled analogs Ile8→ ***L*** **-**CF_3_-Bpg (C), and Ile20→ ***L*** **-**CF_3_-Bpg (D) by HeLa cells. The cells were incubated with 10 µM peptide at 37°C for 30 min.

The cell uptake of the carboxyfluorescein-labeled ***L*** **-**CF_3_-Bpg TP10 analogs was investigated by confocal fluorescence microscopy and compared to the WT peptide. The fluorescence was predominantly localized in punctate structures in the cytoplasm of HeLa cells after incubation for 30 min at 37°C (Figure 2 and Figure S4 in File S1). Since all ***L*** **-**epimers of TP10 showed an activity similar to the WT, they are functionally active and suitable for the subsequent ^19^F-NMR structure analysis.

### Structure Analysis of Monomeric TP10, based on the *L*-epimers at Low Peptide Concentration

**Figure 3:**
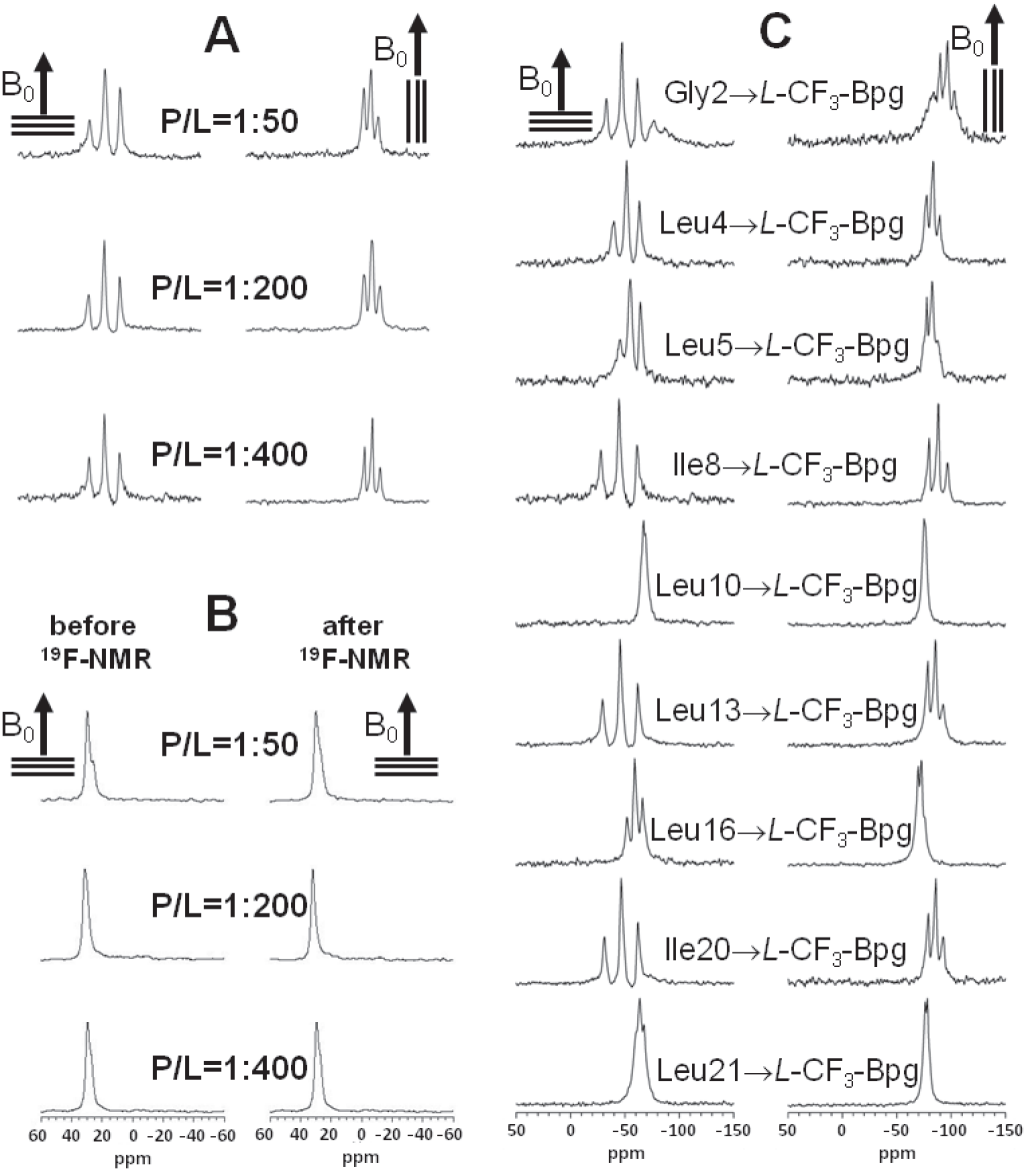
Solid-state NMR spectra of TP10: (A) ^19^F-NMR spectra of TP10 labeled with ***L*** **-**CF_3_-Bpg at Ile8, recorded at three different peptide-to-lipid molar ratios (P/L = 1∶50, 1∶200, and 1∶400) in oriented DMPC/DMPG (3∶1) bilayers. The hydrated membrane samples were aligned with their normal parallel (0°) and perpendicular (90°) to the static magnetic field B_0_ (indicated by an arrow). (B) Solid-state ^31^P-NMR spectra of the same samples as in (A), recorded before and after the corresponding ^19^F-NMR experiment, showing a high quality of lipid alignment. (C) Solid-state ^19^F-NMR spectra of the nine ***L*** **-**CF_3_-Bpg labeled TP10 analogs at P/L = 1∶400, from which the dipolar couplings of the CF_3_-groups were obtained for the structure calculation. All experiments were performed at 40°C.

To determine the three-dimensional structure of membrane-bound TP10, we carried out solid-state ^19^F-NMR experiments in oriented DMPC/DMPG (3∶1) bilayers. The ***L*** **-**epimeric peptides were used for this analysis, as ***L*** **-**CF_3_-Bpg has the same configuration as natural amino acids. This side chain does not usually perturb the peptide conformation when substituted for a bulky hydrophobic residue, as in the case of the labeled positions Leu4, Leu5, Ile8, Leu10, Leu13, Leu16, Ile20, and Leu21, (plus another label at Gly2). The structural compatibility of ***L*** **-**CF_3_-Bpg has been demonstrated above by CD, and was previously shown also for other membrane-active peptides [40], [52]. For the NMR structure analysis we first had to determine and stay below the concentration threshold at which the ***L*** **-**epimeric TP10 analogs would start to aggregate. The unique sensitivity of ^19^F-NMR makes it possible to detect even very low peptide concentrations [49], [56], [80], allowing us to readily screen P/L ratios of 1∶50, 1∶200, and 1∶400 (Figure 3A). Only at 1∶400 did we obtain sharp ^19^F-NMR spectra with no signs of aggregation (as explained below) for all TP10 ***L*** **-**epimers (Figure 3C), even after prolonged sample storage. Under these conditions also the ^31^P-NMR spectra of the phospholipids confirmed a high quality in terms of the degree of lipid orientation in the samples before and after the ^19^F-NMR measurements, with around 85% of the lipids being well-oriented (Figure 3B).

The structure analysis was thus carried out at P/L = 1∶400, where the well-resolved ^19^F-NMR spectra yielded distinct dipolar splittings, and where all ^19^F-labeled analogs can be safely assumed to reflect the monomeric structure of TP10. The absence of aggregation could be further confirmed by showing that the peptides are mobile on the millisecond timescale of the NMR experiment due to free rotational diffusion in the bilayer plane [39], [49], [52], [56]. To examine mobility, each oriented sample was measured twice: with the bilayer normal aligned parallel (0°) and perpendicular (90°) to the direction of the external magnetic field B_0_. As the dipolar splittings at 0° were found to be scaled by a factor of −1/2 upon changing the orientation to 90°, this means that the peptides are engaged in fast rotation around the bilayer normal, which is indicative of monomers or very small oligomers at most [39], [40], [49], [56]. The NMR data of all TP10 ***L*** **-**epimers showed this behavior at low peptide concentration (P/L = 1∶400), confirming that no aggregation had taken place. In some of the NMR spectra at higher peptide concentration (P/L = 1∶50) an emerging powder pattern suggested the onset of immobilization. Yet, even in those cases the sharp dipolar couplings of the mobile component did not change with peptide concentration (Figure S5 and Table S3 in File S1). It can thus be concluded that TP10 has a well-defined structure at low concentration and does not undergo any concentration-dependent re-alignment or gradual conformational change before the onset of aggregation. All NMR spectra at P/L = 1∶50 and 1∶200 are given in Supporting Information (Figure S5 in File S1), and the dipolar couplings at P/L = 1∶50 and 1∶200 are listed in Table S3 in File S1.

**Figure 4:**
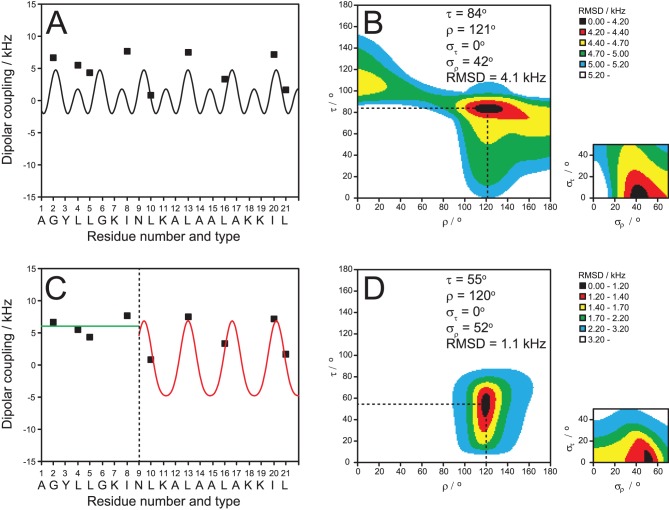
NMR structure analysis of TP10. Dipolar wave analysis of the ^19^F-NMR dipolar couplings of monomeric TP10 in hydrated membranes (DMPC/DMPG, P/L = 1∶400). It is not possible to fit all nine ***L*** **-**CF_3_-Bpg labels to a continuous α-helix (A), as a poor fit with a high RMSD would be obtained (B). A fit of the five C-terminal labels (C, red) produces a good result with a low RMSD (different color scale compared to B) (D). The alignment of the C-terminal α-helix is described by a tilt angle τ≈55° and an azimuthal rotation angle ρ≈120°, with a moderate wobble (σ_ρ_) around the long axis. The black region of the plot shows the possible range of τ and ρ with the same RMSD. The N-terminal region of TP10 is intrinsically unstructured in the plane of the membrane, as seen from the characteristic uniform dipolar splittings of around +7 kHz (C, green).

The well-resolved dipolar splittings of the CF_3_-groups in the nine ***L*** **-**epimers were used as orientational constraints to calculate the three-dimensional structure of monomeric TP10 in the membrane. Details of the corresponding dipolar wave analysis have been described elsewhere [39], [55], [81], [82]. Briefly, if a regular secondary structure such as an α-helix can be presumed, a least-squares fit will yield the helix tilt angle (τ) and the azimuthal rotation angle (ρ), plus the mobility parameters σ_τ_ and σ_ρ_. In the case of TP10, there is ample evidence for an α-helical conformation, based on the CD analysis above and on the liquid-state ^1^H-NMR structure of the parent peptide transportan [12], [13], [47]. However, when we attempted to fit the data of all nine ***L*** **-**CF_3_-Bpg labels to a straight α-helix, this led to an insufficient quality of the fit with a very large root-mean-square deviation (RMSD) (Figures 4A and 4B). On the other hand, it is known that the NMR structure of transportan in SDS micelles and phospholipid bicelles has a kink around Asn15 (equivalent to Asn9 in TP10) and a more flexible N-terminus [12], [13]. Therefore, assuming an helical structure as evidenced by CD spectroscopy, we carried out a segmental analysis of the individual N- and C-terminal parts separately and obtained a very good fit for the C-terminal region with a low RMSD (Figures 4C and 4D).

**Figure 5:**
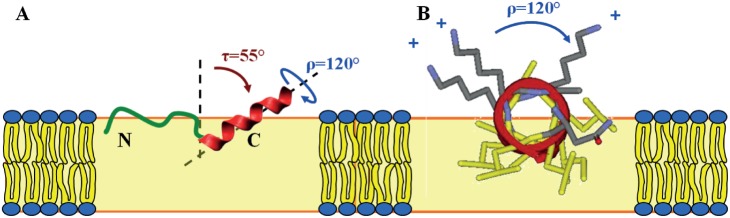
Membrane-bound structure of TP10, as derived by solid-state ^19^F -**NMR**. (A) The N-terminal region is intrinsically unstructured (green) and connected to the C-terminal α-helix (red). The amphiphilic helix is embedded in the lipid membrane with a tilt angle of τ≈55° and an azimuthal rotation angle of ρ≈120°. (B) The helical wheel projection of the C-terminal mastoparan part illustrates how the charged Lys residues (grey) point towards the aqueous phase, while the hydrophobic residues (yellow) face the interior of the membrane. The yellow box represents the bilayer (not drawn to scale, and without implying any particular insertion depth of the peptide within the bilayer).

These data suggest that the C-terminal region of TP10, which was derived from mastoparan, has a well-defined α-helical fold in the membrane-bound state. This finding supports our observation by CD that membrane-bound TP exhibits roughly 56% helicity (Table 1), which would corresponds well to the 12 C-terminal residues. Based on the dipolar couplings of the labeled positions Leu10, Leu13, Leu16, Ile20, and Leu21, the data analysis yielded a helix tilt angle of τ≈55° and an azimuthal rotation angle of ρ≈120°, with a moderate wobble (σ_ρ_) around the long axis. The alignment of the α-helix in the lipid bilayer is illustrated in Figure 5A. The obtained azimuthal rotation angle is in full agreement with the amphiphilic character of the helix. The helical wheel projection of the membrane-embedded C-terminal region in Figure 5B shows that the hydrophobic residues face the bilayer interior, while the positively charged Lys residues point towards the aqueous layer.

In contrast, the dipolar splittings of the N-terminal part of TP10 could not be fitted to an α-helix, or to any other regular secondary structure element. Instead, all dipolar couplings of the N-terminal labels (positions Gly2, Leu4, Leu5, Ile8) are close to +7 kHz, a value that has been reported as characteristic for a unstructured peptide backbone that is swimming around flexibly in the membrane [40]. A previous study on isolated galanin peptides had also shown that this glycine-rich sequence is largely unstructured in various environments, including lipid bilayers and detergent micelles [83]. We can thus refer to the galanin-derived segment of TP10 as a region of the peptide that is intrinsically unstructured within the two-dimensional membrane plane - analogous to the concept of intrinsically unstructured proteins in aqueous solution [84], [85]. We thus conclude that the galanin-derived N-terminus of TP10 is essentially unstructured within the 2D plane of the membrane, while being anchored to the bilayer surface by its hydrophobic residues. The detailed structure derived here by ^19^F-NMR is good agreement with the qualitative picture obtained above by CD lineshape deconvolution (see Table 1), for which the estimated 56% helical fold can be attributed now to the C-terminal mastoparan-derived segment.

### Analysis of the Aggregation Behavior of TP10, based on the *D*-epimers

Next, the dipolar splittings were measured for the series of TP10 epimers labeled with ***D*** **-**CF_3_-Bpg, where the C_α_ configuration is stereochemically inverted. At a low peptide concentration of P/L = 1∶400, the splittings along the entire sequence are all rather close to +7 kHz (Table S4 in File S1). The C-terminal part of TP10 can clearly no longer be fitted to an α-helix. Instead, the characteristic range of dipolar splittings suggests that the helix has now become unfolded within the plane of the membrane, just like the N-terminus. It thus appears that the introduction of a single bulky ***D*** **-**CF_3_-Bpg residue in the C-terminal segment leads to local destabilization around the ***D*** **-**amino acid and possibly of the entire α-helical part. On the other hand, incorporation of ***D*** **-**CF_3_-Bpg in the N-terminal region does not have any significant structural consequences, as this part of the peptide is intrinsically flexible anyway.

**Figure 6:**
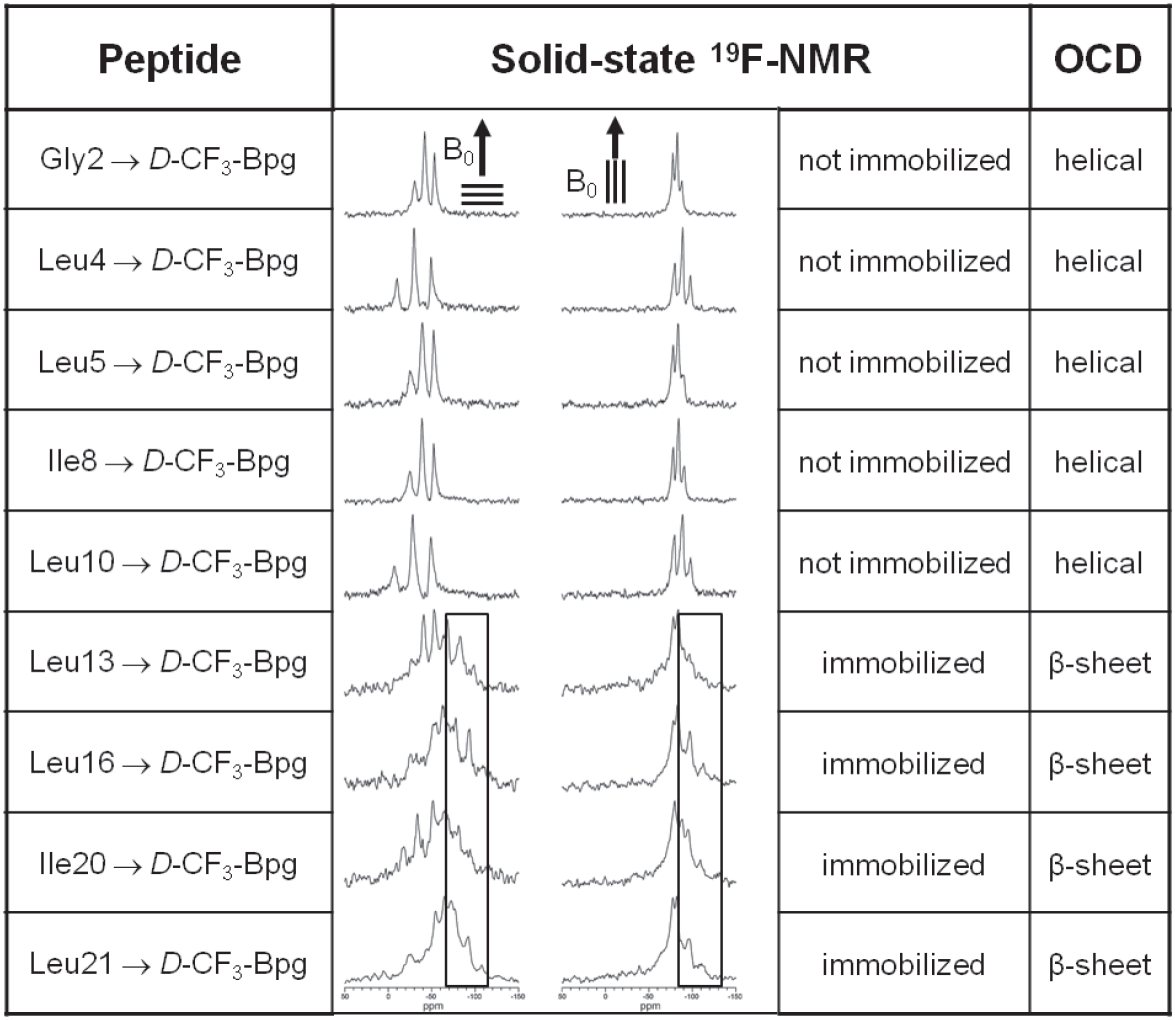
*D*-amino acid “scan” to identify aggregation-prone regions in TP10. Aggregation of TP10 depends on the position of substitution with the sterically restrictive ***D*** **-**CF_3_-Bpg, as monitored by solid-state ^19^F-NMR and OCD in oriented DMPC/DMPG (3∶1) at P/L = 1∶50. The boxed spectral regions show the static powder pattern contributions of immobilized molecules with −8 kHz splittings.

Having found that ***D*** **-**CF_3_-Bpg destabilizes the helical region of TP10 and leads to unfolding in the membrane at low concentration, we also examined the ***D*** **-**epimers at a high P/L ratio of 1∶50. The ^19^F-NMR spectra of freshly prepared samples are shown in Figure 6, and the splittings are listed in Table S4 in File S1. It appears that all analogs with ***D*** **-**substituents in the C-terminal region of TP10 (at positions Leu13, Leu16, Ile20, or Leu21) are partly or completely aggregated. This is seen from the static powder components in the lineshapes, with dipolar splittings of −8 kHz (as illustrated by the boxes in Figure 6). Upon changing the sample alignment from 0° to 90° these splittings are no longer reduced by a factor of −1/2, which is a sign that the peptide molecules are completely immobilized. On the other hand, introduction of ***D*** **-**CF_3_-Bpg into the N-terminal region of TP10 (at position Gly2, Leu4, Leu5, Ile8, or Leu10) did not lead to aggregation. In these cases, all dipolar splittings remained close to +7 kHz as seen for P/L = 1∶400, and they were reduced by a factor of −1/2 when the sample alignment was changed from 0° to 90° (see Figure 6, and Table S4 in File S1). It may thus be concluded that a destabilization of the α-helical mastoparan part of TP10 by the sterically restrictive ***D*** **-**CF_3_-Bpg leads to unfolding at low peptide concentration and an enhanced aggregation at high concentration.

**Figure 7:**
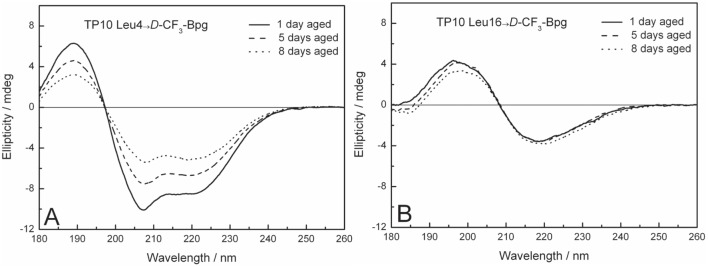
OCD spectra of TP10. Representative OCD spectra of TP10 labeled with ***D*** **-**CF_3_-Bpg in oriented DMPC/DMPG (3∶1) bilayers at P/L = 1∶50, measured after 1, 5, and 8 days of ageing. (A) Peptide analogs with a substitution in the N-terminal region (here: position Leu4) have a predominantly α-helical structure, just like the WT peptide. (B) When ***D*** **-**CF_3_-Bpg is placed into the C-terminal region (here: position Leu16), the peptide aggregates with a β-sheet conformation typical of amyloid-like fibrils.

Strictly speaking, the appearance of a static ^19^F-NMR powder pattern does not prove aggregation *per se*, but it shows that the molecules are no longer well-oriented in the membrane and are immobilized on the millisecond time-scale of the NMR experiment. It is impossible to deduce any structural parameters from these powder spectra, because all orientational order has been lost. We thus wanted to find out whether the immobilized peptides (P/L = 1∶50 positions Leu10, Leu13, Leu16, IIe20, Leu21 in Table S4 in File S1) have taken on a characteristic β-sheet conformation, as often implied for aggregation and amyloid formation. To this aim, we employed oriented circular dichroism (OCD) to determine the conformation of the TP10 analogs in the same type of oriented DMPC/DMPG (3∶1) samples and at the same P/L ratio of 1∶50 as in ^19^F-NMR. To pick up any slow kinetics of aggregation, ageing studies were performed over 1, 5 and 8 days of incubation at 48°C under a fully hydrated atmosphere, yielding the characteristic OCD spectra illustrated in Figure 7. The complete set of OCD spectra for the WT peptide and all the ***D*** **-**epimers is presented in Figure S6 in File S1. The observed decrease in spectral intensity with time is attributed to lateral phase segregation of the membrane-bound helical peptides into peptide-rich domains, which reduces the intensity of the CD signal.

OCD analysis demonstrated that all TP10 analogs with a ***D*** **-**CF_3_-Bpg substitution in the C-terminal region gave typical β-sheet lineshapes (Figure 7B). On the other hand, substitutions in the N-terminal part had no impact on the predominantly α-helical conformation of the ***D*** **-**epimeric peptides (Figure 7A), which behaved like the TP10 WT. We thus conclude that incorporation of the bulky ***D*** **-**amino acid into the flexible N-terminal region did not disturb the peptide structure and preserved the folded helical C-terminus. In contrast, ***D*** **-**CF_3_-Bpg in the C-terminal region led to an unfolding of the helix and made the peptide more disordered at low concentration, which permitted its aggregation at higher concentration into β-sheets. Overall, we have thus identified by OCD the same pattern of aggregation as seen by solid-state ^19^F-NMR under the same conditions in the same type of samples.

Transportan, like many other membrane-active peptides, has a known tendency to aggregate at high concentration [28], [29], [32], [35]–[37]. In the case of TP10, we recall that several ***L*** **-**epimeric TP10 analogs had already shown signs of aggregation at P/L = 1∶50 in fresh ^19^F-NMR samples (Figure S5 in File S1). The aggregation process is typically surface-induced and accelerated in the membrane-bound state as reported for various systems [35], [86]–[88], where all peptide molecules are confined within the two-dimensional plane of the bilayer with an increased local concentration. Aggregation is often associated with the transition of an intrinsically unstructured peptide into oligomeric β-sheets in a concentration and time dependent manner. We have recently used the model peptide [KIGAKI]_3_ to examine this process and characterize its typical ^19^F-NMR signature [40], [89]. Self-assembly of this system in membranes [40], [66] leads to formation of fibrils that are similar to amyloid consisting of hydrogen-bonded cross-β-strands [32]–[34], [36], [90].

**Figure 8:**
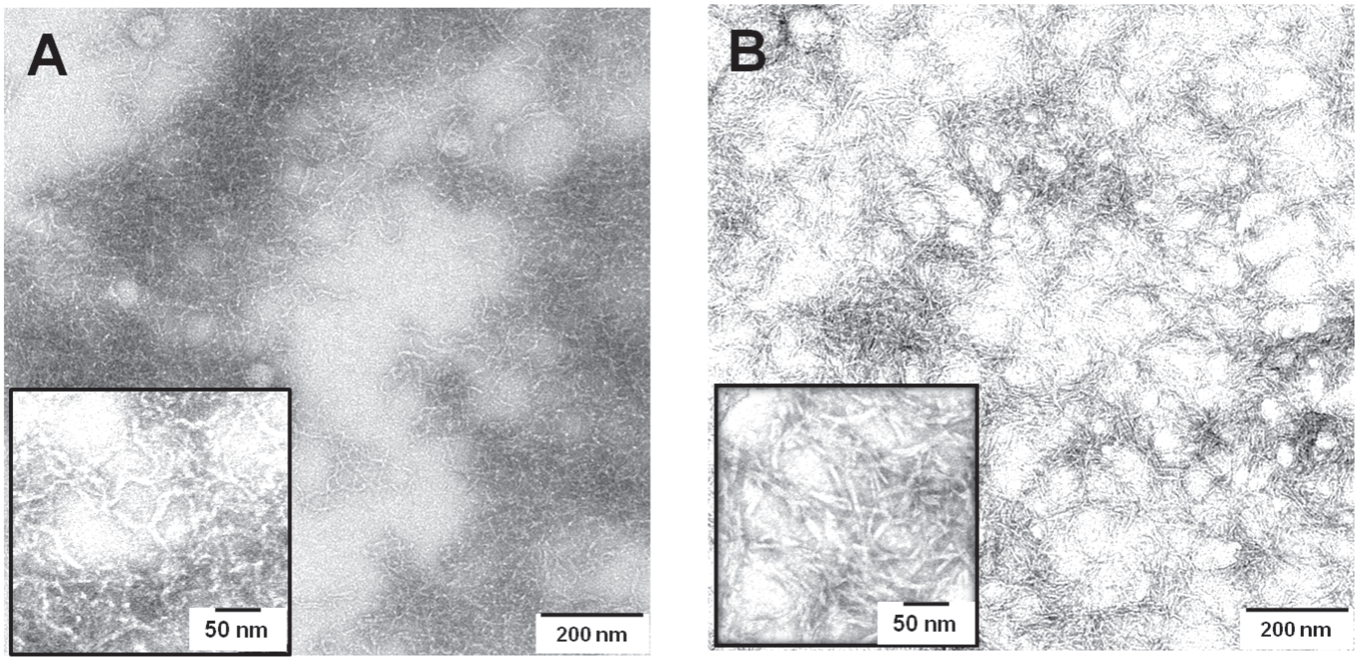
Fibril formation of TP10. TEM images of TP10 analogs (A) Leu16→ ***L*** **-**CF_3_ *-*Bpg, (B) Leu16→ ***D*** **-**CF_3_-Bpg, showing a network of amyloid-like fibrils.

It is intrinsically difficult to prove the formation of genuine amyloid fibrils in the presence of membranes. For example, the thioflavin-T assay does not work, because the positive charge on the thioflavin-T micelles interferes with binding to the cationic peptides due to electrostatic repulsion [91]. Electron microscopy in the presence of membranes is problematic, too, because mixtures of lipids with self-assembling peptides can give rise to unusual morphologies in which it is hard to discriminate regular fibrils [92]. Therefore, we decided to use TEM to directly observe the peptide aggregates in the absence of any lipid. The ***L*** **-** and ***D*** **-**epimers labeled at position Leu16 were incubated in water at 2 mM concentration at 20°C. After 24 hours the sample with the ***L*** **-**epimer was opaque and the ***D*** **-**epimer even more so. The representative TEM images in Figure 8 reveal a network of short fibrils in both samples, reminiscent of amyloid fibrils, thus supporting the OCD observations above (for OCD of ***L*** **-**epimer see Figure S7 in File S1). Assuming that peptide aggregation is an intrinsic property of a given sequence and that its immediate environment influences mainly the kinetics of aggregation but not the local morphology, it is reasonable to argue that the observed cross-β-sheet fibrils in the absence of lipids should be similar to those in the presence of membranes.

## Discussion

**Figure 9:**
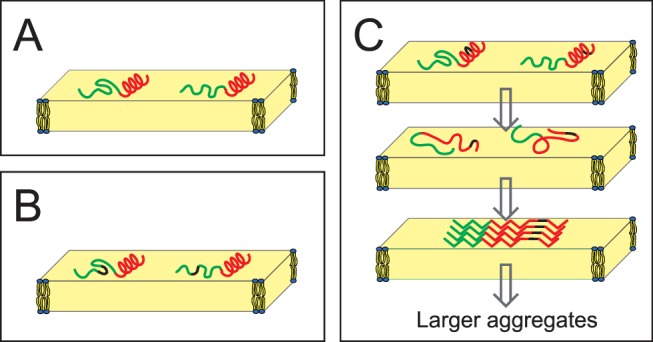
Structure and aggregating behavior of TP10. (A) Monomeric structure of TP10 as determined from ^19^F-NMR in the membrane-bound state. The N-terminal galanin-derived region (green) is intrinsically unstructured, while the mastoparan-derived C-terminus (red) is folded as an α-helix. (B) When ***D*** **-**CF_3_-Bpg (black) is introduced into the flexible region, there is no conformational change. (C) However, when this stiff ***D*** **-**amino acid is placed into the helical C-terminal region, it leads to unfolding. At low concentration the monomers remain unstructured, but at high concentration surface-induced aggregation leads to the assembly of β-pleated amyloid-like fibrils. These cross-β-sheet assemblies must be distorted in the immediate vicinity of the ***D*** **-**amino acid. There is no indication if the β-sheets are parallel or antiparallel. As the β-sheets have no preferential orientation in the lipid bilayer (and they may be twisted), they give powder-type NMR spectra.

We have shown that TP10 can assume three distinctly different conformations in the membrane-bound state (Figure 9). The monomeric structure has a well-defined α-helix in the C-terminal region, and a flexible N-terminus that may be regarded as intrinsically unstructured in the plane of the membrane (Figure 9A). This picture is consistent with an earlier NMR analysis in detergent micelles [47], and with a more recent MD study of TP10 in a POPC bilayer [93]. If the helix is perturbed, the peptide unfolds completely and starts to aggregate as amyloid-like fibrils in a concentration and time dependent manner. While the incorporation of ***L*** **-**CF_3_-Bpg as a ^19^F-NMR label does not significantly perturb the peptide, the sterically obstructive ***D*** **-**epimer can be used to dramatically shift the transition from the partially α-helical monomer ↔ unfolded monomer ↔ β-pleated aggregate. Remarkably, the tendency to aggregate depends distinctly on the position of the substitution. When ***D*** **-**CF_3_-Bpg is incorporated into the intrinsically flexible N-terminal region of TP10, the peptide maintains its usual partially α-helical structure (Figure 9B). A substitution in the C-terminal region, on the other hand, leads to unfolding and subsequent aggregation as β-sheets at high concentration (Figure 9C).

The effect of ***D*** **-**CF_3_-Bpg on TP10 resembles the reported destabilization of the α-helical region in the Aβ peptide by ***D*** **-**amino acids or proline, which subsequently leads to enhanced aggregation [94]. At the same time - and seemingly contradictory at first sight - it has been reported that ***D*** **-**amino acids can also be used to prevent the aggregation of peptides [39], [40], [89]. This effect is not attributed to equilibrium between helix and unfolded state (as in TP10 or Aβ), but instead to the self-assembly of already unfolded peptides (from the β-stranded KIGAKI family) into aggregated fibrils. The resulting β-sheets are destabilized, because the sterically obstructive side chain cannot be readily accommodated in the ordered core of the amyloid. In the case of a sensitive helix-to-sheet equilibrium, the decisive aspect is whether the α-helix or the β-sheet is more strongly perturbed by the ***D***-amino acid. Along the same lines, it has been reported that the systematic replacement of amino acid pairs with ***D***-ProGly could accelerate Aβ fibril formation [95], while pairs of ***D*** **-**amino acids, or even single proline residues, have been used as amyloid breakers [96]. It was thus interesting to see that not only the ***L*** **-** but also the ***D*** **-**epimers of TP10 ended up as amyloid-like fibrils. To reconcile these observations, it must be noted that β-sheets can actually tolerate the odd misfit. For example, the β-pleated model peptide [KIGAKI]_3_ can assemble as β-sheets even with one third of its amino acids exchanged to the ***D*** **-**form [65], [66], meaning that only the ***D*** **-**amino acid itself and its closest neighbors are excluded from the ordered array of hydrogen-bonds in a typical sheet. It is therefore perfectly reasonable that a bulky ***D*** **-**amino acid can have both effects, of either preventing or promoting amyloid formation. The observed outcome depends on the *relative* destabilization of the original (α-helical) conformation compared to the resulting β-sheet structure. In the case of TP10, which already contains an unstructured N-terminus, the aggregation equilibrium is shifted to the right when the destabilizing ***D*** **-**CF_3_-Bpg is incorporated into the C-terminus. In the Model Amphiphilic Peptide MAP [KLALKLALKALKAALKLA-NH_2_], on the other hand, the equilibrium is shifted to the left and aggregation is prevented by a ***D*** **-**amino acid. That is because MAP can engage in favorable interactions with the membrane only as an amphiphilic helix [39], [97]. Likewise, in [KIGAKI]_3_, bulky ***D*** **-**amino acids shift the aggregation threshold to higher peptide concentration, as the unfolded peptide has no reason to become α-helical [40], [65], [66].

**Figure 10:**
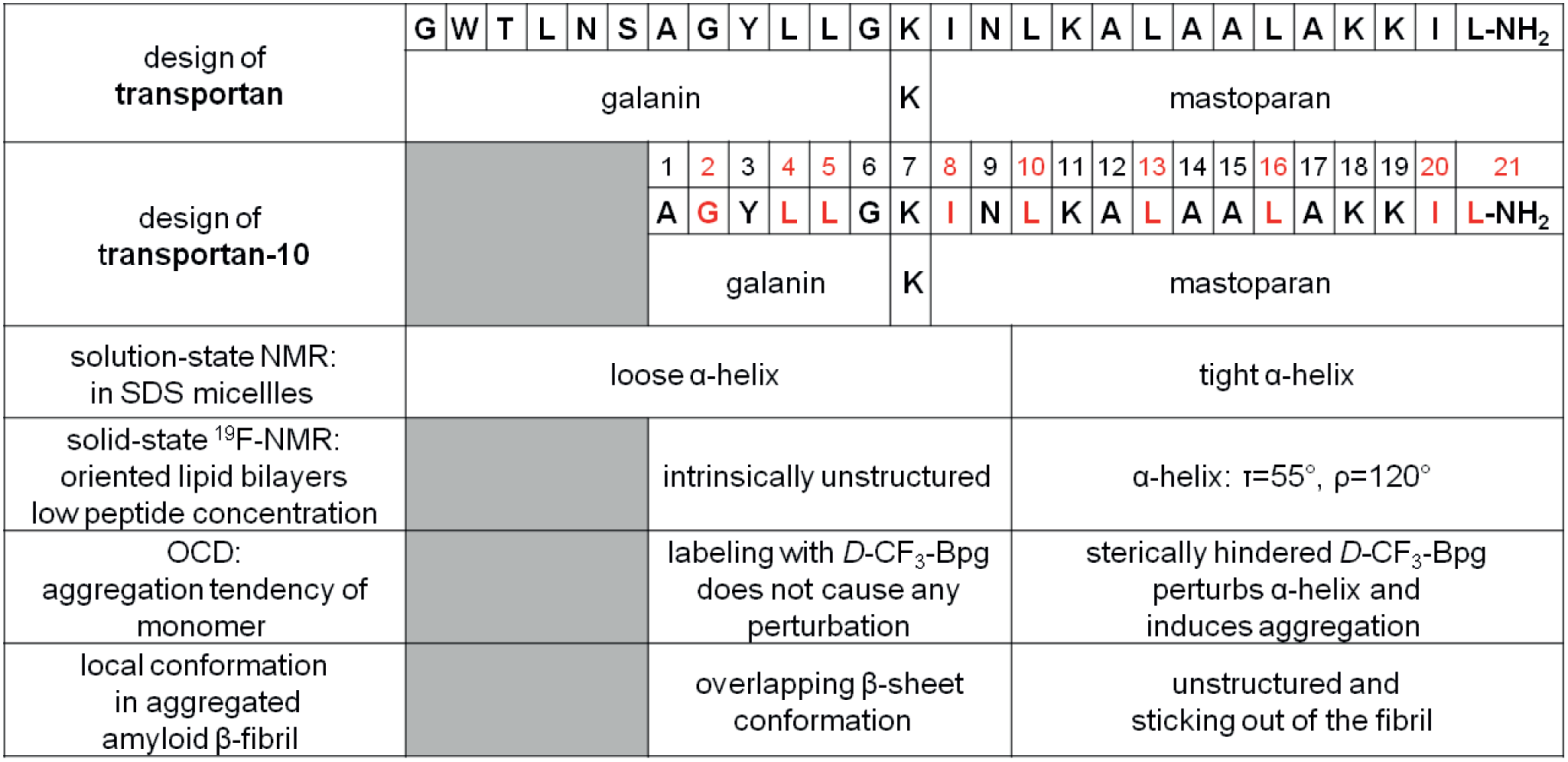
Structural characteristics of TP10. Summary of features related to the bipartite character of the hybrid peptide TP10 (positions labeled with CF_3_-Bpg are marked in red).

Based on the TEM images and the OCD spectra of aggregated TP10 with ***D*** **-**CF_3_-Bpg, which show an essentially complete β-structure, we expect that the entire peptide may assume a regular β-sheet conformation in the presence as well as absence of membranes, apart from the local position that is labeled with ***D*** **-**CF_3_-Bpg and its immediate surroundings. There is of course no indication from our data on the parallel or antiparallel alignment, or any possible staggering. However, a parallel unstaggered arrangement as depicted in Figure 9C would minimize the destabilizing effects of the ***D*** **-**amino acid on the β-sheet and is therefore most likely. We trust that this picture offers for the first time an appropriate description of (a) the monomeric structure of TP10 in a lipid bilayer, and (b) of its possible conformational switch in the presence of a cellular membrane at high concentration. To our knowledge this is the first detailed study focusing on the structure analysis and aggregation propensity of a cell-penetrating peptide in its functionally relevant membrane-bound state. TP10 is a truncated derivative of transportan, a designer-made hybrid that was constructed from an N-terminal galanin sequence and a C-terminal mastoparan part, as illustrated in Figure 10. Transportan and several other CPPs have been previously characterized in detergent micelles and/or bicelles [12], [47], [98], [99], but the oriented lipid bilayers used here for solid-state ^19^F-NMR and OCD analysis resemble native cell membranes much more closely. Our comprehensive structure analysis of TP10 has confirmed its intrinsic bipartite character (Figure 10) and refined the structure of the peptide in genuine lipid bilayers. It is remarkable to see that the underlying sequences and presumably the conformational features of the original galanin and mastoparan building blocks are essentially retained in the hybrid peptide. TP10 contains a glycine-rich, unstructured flexible N-terminal region, and a C-terminal α-helix that is obliquely tilted in the membrane in accordance with its amphiphilic profile (Figure 5). Besides having resolved the biologically relevant peptide conformation, our results have also yielded the distinct membrane alignments of the individual peptide segments and explained their role in aggregation. Being a typical membrane-active peptide, this structural description of TP10 is relevant for the wide group of cell-penetrating and antimicrobial peptides in general, and it contributes also to a better understanding of lipid-induced amyloid fibril formation.

## Supporting Information

File S1 Figure S1, CD spectra of the CF_3_-Bpg labeled TP10 analogs bound to DMPC/DMPG vesicles. (A) *L*- and (B) *D*-epimers at P/L = 1∶50. (C) *L*- and (D) *D*-epimers at P/L = 1∶100. The wild type TP10 is shown as a black line; green lines show analogs with CF_3_-Bpg labels in the galanin part, and red lines in the mastoparan part. Figure S2, CD spectra of the carboxyfluorescein- and CF_3_-Bpg labeled TP10 analogs in DMPC/DMPG vesicles at P/L = 1∶50. (A) *L*- and (B) *D*-epimers. The wild type TP10 is shown as a black line; green lines show analogs with CF_3_-Bpg labels in the galanin part, and red lines in the mastoparan part. Figure S3, Comparison of the solid-state ^19^F-NMR spectra of *L*-CF_3_-Bpg labeled TP10. Analogs with and without the carboxyfluorescein-label were measured in oriented DMPC/DMPG bilayers at P/L = :200, at 40°C, with the sample normal aligned parallel (0°) and perpendicular (90°) to the static magnetic field. Figure S4, Internalization of the carboxyfluorescein-labeled TP10-WT and the *L*- and *D*-CF_3_-Bpg analogs by HeLa-cells. The cells were incubated with 2 µM (left column) and 10 µM (right column) peptide at 37°C for 30 min. Figure S5, Solid-state ^19^F-NMR spectra of the *L*-CF_3_-Bpg labeled TP10. Analogs were measured in oriented DMPC/DMPG bilayers at P/L = 1∶50 and 1∶200, at 40°C, with the sample normal aligned parallel (0°) and perpendicular (90°) to the static magnetic field B_0_. Several spectra showed an immobilized powder component, as indicated by the boxes. Figure S6, OCD spectra of the *D*-CF_3_-Bpg labeled TP10. Analogs were measured in oriented DMPC/DMPG bilayers at P/L = 1∶50. Spectra were recorded after 1 (straight lines), 5 (dashed lines) and 8 days (dotted lines) hydration of the OCD sample. Figure S7, OCD spectra of the Leu16→*L*-CF_3_-Bpg labeled TP10. Peptide measured in oriented DMPC/DMPG bilayers at P/L = 1∶50. Spectra were recorded after 1 (straight lines), 5 (dashed lines) and 8 days (dotted lines) hydration of the OCD sample. Scheme S1, Chemical structure of *L*-CF_3_-Bpg (left) and *D*-CF_3_-Bpg (right). Table S1, Sequences and LC-MS characterization of TP10-WT and the analogues labeled with *L*-CF_3_-Bpg and with an additional carboxyfluorescein (CF). (Equivalent data were obtained for the *D*-epimers). Table S2, Comparison of the ^19^F-NMR dipolar couplings of the *L*-CF_3_-Bpg labeled TP10 analogs with and without the fluorescent carboxyfluorescein label in oriented DMPC/DMPG bilayers (P/L = 1∶200), measured at 0° and 90° sample tilt. Table S3, Dipolar couplings of the *L*-CF_3_-Bpg labeled TP10 analogs in oriented DMPC/DMPG bilayers, measured at 0° and 90° sample tilt, at P/L = 1∶50, 1∶200, and 1∶400. Table S4, Dipolar couplings of the *D*-CF_3_-Bpg labeled TP10 analogs in oriented DMPC/DMPG bilayers, measured at 0° and 90° sample tilt, at P/L = 1∶50, 1∶200, and 1∶400.
(DOCX)
