# Supplementary material for: Structure Analysis and Conformational Transitions of the Cell Penetrating Peptide Transportan 10 in the Membrane-Bound State
Source: PLoS One. 2014 Jun 17;9(6):e99653. doi: 10.1371/journal.pone.0099653 (PMC4061077; doi:10.1371/journal.pone.0099653)
Supplement: File S1 — Figure S1, CD spectra of the CF3-Bpg labeled TP10 analogs bound to DMPC/DMPG vesicles. (A) L- and (B) D-epimers at P/L = 1∶50. (C) L- and (D) D-epimers at P/L = 1∶100. The wild type TP10 is shown as a black line; green lines show analogs with CF3-Bpg labels in the galanin part, and red lines in the mastoparan part. Figure S2, CD spectra of the carboxyfluorescein- and CF3-Bpg labeled TP10 analogs in DMPC/DMPG vesicles at P/L = 1∶50. (A) L- and (B) D-epimers. The wild type TP10 is shown as a black line; green lines show analogs with CF3-Bpg labels in the galanin part, and red lines in the mastoparan part. Figure S3, Comparison of the solid-state 19F-NMR spectra of L-CF3-Bpg labeled TP10. Analogs with and without the carboxyfluorescein-label were measured in oriented DMPC/DMPG bilayers at P/L = :200, at 40°C, with the sample normal aligned parallel (0°) and perpendicular (90°) to the static magnetic field. Figure S4, Internalization of the carboxyfluorescein-labeled TP10-WT and the L- and D-CF3-Bpg analogs by HeLa-cells. The cells were incubated with 2 µM (left column) and 10 µM (right column) peptide at 37°C for 30 min. Figure S5, Solid-state 19F-NMR spectra of the L-CF3-Bpg labeled TP10. Analogs were measured in oriented DMPC/DMPG bilayers at P/L = 1∶50 and 1∶200, at 40°C, with the sample normal aligned parallel (0°) and perpendicular (90°) to the static magnetic field B0. Several spectra showed an immobilized powder component, as indicated by the boxes. Figure S6, OCD spectra of the D-CF3-Bpg labeled TP10. Analogs were measured in oriented DMPC/DMPG bilayers at P/L = 1∶50. Spectra were recorded after 1 (straight lines), 5 (dashed lines) and 8 days (dotted lines) hydration of the OCD sample. Figure S7, OCD spectra of the Leu16→L-CF3-Bpg labeled TP10. Peptide measured in oriented DMPC/DMPG bilayers at P/L = 1∶50. Spectra were recorded after 1 (straight lines), 5 (dashed lines) and 8 days (dotted lines) hydration of the OCD sample. Scheme S1, Chemical structure of L-CF [file pone.0099653.s001.docx]

**Supporting information**

**Structure analysis and conformational transitions of the cell penetrating peptide transportan 10 in the membrane-bound state**

Susanne Fanghänel,^†,‡^ Parvesh Wadhwani,^§,‡^ Erik Strandberg,^§^ Wouter P. R. Verdurmen,**^‖^** Jochen Bürck,^§^ Sebastian Ehni,^†^ Pavel K. Mykhailiuk,**^┴^** Sergii Afonin,^§^ Dagmar Gerthsen,^#^ Igor V. Komarov,^¶^ Roland Brock,**^‖^** Anne S. Ulrich*^,†,§^

^‡^Susanne Fanghänel and Parvesh Wadhwani contributed equally to this work.

^†^Karlsruhe Institute of Technology (KIT), Institute of Organic Chemistry and DFG-Center for Functional Nanostructures (CFN), Karlsruhe, Germany

^§^KIT, Institute of Biological Interfaces (IBG2), Karlsruhe, Germany

**^‖^**Department of Biochemistry, Radboud Institute for Molecular Life Sciences, Radboud University Medical Centre, Nijmegen, The Netherlands

**^┴^**Taras Shevchenko National University of Kyiv, Chemistry Department, Kyiv, Ukraine and Enamine Ltd., Kyiv, Ukraine

^#^KIT, Laboratory for Electron Microscopy, Karlsruhe, Germany

^¶^Taras Shevchenko National University of Kyiv, Institute of High Technologies, Kyiv, Ukraine

**TABLES AND FIGURES**

Table S1: Sequences and LC-MS characterization of TP10-WT and the analogues labeled with *L*-CF_3_-Bpg and with an additional carboxyfluorescein (CF). (Equivalent data were obtained for the *D*-epimers).

| **Peptide** | **Sequence** | **Mass**  **calculated** | **Mass**  **found** |
| --- | --- | --- | --- |
| TP10-WT | AGYLLGKINLKALAALAKKIL-NH_2_ | 2182 | 2183 |
| Gly2 → *L*-CF_3_-Bpg | A **CF_3_-Bpg** YLLGKINLKALAALAKKIL-NH_2_ | 2316 | 2316 |
| Leu4 → *L*-CF_3_-Bpg | AGY **CF_3_-Bpg** LGKINLKALAALAKKIL-NH_2_ | 2260 | 2260 |
| Leu5 → *L*-CF_3_-Bpg | AGYL **CF_3_-Bpg** GKINLKALAALAKKIL-NH_2_ | 2260 | 2260 |
| Ile8 → *L*-CF_3_-Bpg | AGYLLGK **CF_3_-Bpg** NLKALAALAKKIL-NH_2_ | 2260 | 2260 |
| Leu10 → *L*-CF_3_-Bpg | AGYLLGKIN **CF_3_-Bpg** KALAALAKKIL-NH_2_ | 2260 | 2260 |
| Leu13 → *L*-CF_3_-Bpg | AGYLLGKINLKA **CF_3_-Bpg** AALAKKIL-NH_2_ | 2260 | 2261 |
| Leu16 → *L*-CF_3_-Bpg | AGYLLGKINLKALAA **CF_3_-Bpg** AKKIL-NH_2_ | 2260 | 2260 |
| Ile20 → *L*-CF_3_-Bpg | AGYLLGKINLKALAALAKK **CF_3_-Bpg** L-NH_2_ | 2260 | 2260 |
| Leu21 → *L*-CF_3_-Bpg | AGYLLGKINLKALAALAKKI **CF_3_-Bpg** -NH_2_ | 2260 | 2260 |
|  |  |  |  |
| CF-TP10-WT | **CF**-AGYLLGKINLKALAALAKKIL-NH_2_ | 2540 | 2541 |
| Gly2 → CF-*L*-CF_3_-Bpg | **CF**-A **CF_3_-Bpg** YLLGKINLKALAALAKKIL-NH_2_ | 2674 | 2675 |
| Leu4 → CF-*L*-CF_3_-Bpg | **CF**-AGY **CF_3_-Bpg** LGKINLKALAALAKKIL-NH_2_ | 2618 | 2619 |
| Leu5 → CF-*L*-CF_3_-Bpg | **CF**-AGYL **CF_3_-Bpg** GKINLKALAALAKKIL-NH_2_ | 2618 | 2619 |
| Ile8 → CF-*L*-CF_3_-Bpg | **CF**-AGYLLGK **CF_3_-Bpg** NLKALAALAKKIL-NH_2_ | 2618 | 2619 |
| Leu10 → CF-*L*-CF_3_-Bpg | **CF**-AGYLLGKIN **CF_3_-Bpg** KALAALAKKIL-NH_2_ | 2618 | 2619 |
| Leu13 → CF-*L*-CF_3_-Bpg | **CF**-AGYLLGKINLKA **CF_3_-Bpg** AALAKKIL-NH_2_ | 2618 | 2619 |
| Leu16 → CF-*L*-CF_3_-Bpg | **CF**-AGYLLGKINLKALAA **CF_3_-Bpg** AKKIL-NH_2_ | 2618 | 2619 |
| Ile20 → CF-*L*-CF_3_-Bpg | **CF**-AGYLLGKINLKALAALAKK **CF_3_-Bpg** L-NH_2_ | 2618 | 2619 |
| Leu21 → CF-*L*-CF_3_-Bpg | **CF**-AGYLLGKINLKALAALAKKI **CF_3_-Bpg** -NH_2_ | 2618 | 2619 |

**Table S2:** **Comparison of the ^19^F-NMR dipolar couplings of the *L*-CF_3_-Bpg labeled TP10 analogs with and without the fluorescent carboxyfluorescein label in oriented DMPC/DMPG bilayers (P/L=1:200), measured at 0° and 90° sample tilt**.

| **Peptide** | **Dipolar coupling [kHz]** | | | |
| --- | --- | --- | --- | --- |
|  | Without CF-label | | With CF-label | |
|  | 0° | 90° | 0° | 90° |
| Gly2 → *L*-CF_3_-Bpg* | -7.5 | -7.5 | na** | na** |
| Leu4 → *L*-CF_3_-Bpg | 5.6 | -2.6 | 6.8 | -3.4 |
| Leu5 → *L*-CF_3_-Bpg | 4.9 | -2.4 | 1.1 | 0.7 |
| Ile8 → *L*-CF_3_-Bpg | 7.9 | -4.1 | 9.3 | -4.3 |
| Leu10 → *L*-CF_3_-Bpg | 0.9 | -0.4 | 0.9 | -0.4 |
| Leu13 → *L*-CF_3_-Bpg | 7.6 | -3.6 | 8.3 | -4.0 |
| Leu16 → *L*-CF_3_-Bpg | 3.3 | -1.6 | 3.3 | -1.6 |
| Ile20 → *L*-CF_3_-Bpg | 7.3 | -3.4 | 7.6 | -3.5 |
| Leu21 → *L*-CF_3_-Bpg | 2.0 | -1.1 | 2.7 | -0.9 |

* unlike all other analogs, the peptide labeled at Gly2 was not rotationally mobile

** not accessible

**Table S3:** **Dipolar couplings of the *L*-CF_3_-Bpg labeled TP10 analogs in oriented DMPC/DMPG bilayers, measured at 0° and 90° sample tilt, at P/L=1:50, 1:200, and 1:400.**

| **Peptide** | **Dipolar coupling [kHz]** | | | | | | | | |
| --- | --- | --- | --- | --- | --- | --- | --- | --- | --- |
| **P/L ratio** | | **1:50** | | | **1:200** | | **1:400** | | |
| **Sample tilt** | **0°** | | **90°** | **0°** | | **90°** | | **0°** | **90°** |
| Gly2 → *L*-CF_3_-Bpg | -7.5 | | -7.5 | -7.5 | | -7.5 | | 6.7 | -3.1 |
| Leu4 → *L*-CF_3_-Bpg | 5.5 | | -2.7 | 5.6 | | -2.6 | | 5.5 | -2.8 |
| Leu5 → *L*-CF_3_-Bpg | 4.5 | | -2.1 | 4.9 | | -2.4 | | 4.4 | -2.3 |
| Ile8 → *L*-CF_3_-Bpg | 7.8 | | -3.7 | 7.9 | | -4.1 | | 7.8 | -4.0 |
| Leu10 → *L*-CF_3_-Bpg | 0.6 | | -0.2 | 0.9 | | -0.4 | | 0.9 | -0.4 |
| Leu13 → *L*-CF_3_-Bpg | -7.5 | | -7.5 | 7.6 | | -3.6 | | 7.6 | -3.3 |
| Leu16 → *L*-CF_3_-Bpg | -7.5 | | -7.5 | 3.3 | | -1.6 | | 3.4 | -1.4 |
| Ile20 → *L*-CF_3_-Bpg | 6.7 | | -2.9 | 7.3 | | -3.4 | | 7.2 | -3.2 |
| Leu21 → *L*-CF_3_-Bpg | 1.9 | | -1.0 | 2.0 | | -1.1 | | 1.8 | -0.9 |

**Table S4:** **Dipolar couplings of the *D*-CF_3_-Bpg labeled TP10 analogs in oriented DMPC/DMPG bilayers, measured at 0° and 90° sample tilt, at P/L=1:50, 1:200, and 1:400.**

| **Peptide** | **Dipolar coupling [kHz]** | | | | | |
| --- | --- | --- | --- | --- | --- | --- |
| **P/L ratio** | **1:50** | | **1:200** | | **1:400** | |
| **Sample tilt** | **0°** | **90°** | **0°** | **90°** | **0°** | **90°** |
| Gly2 → *D*-CF_3_-Bpg | 5.3 | -2.4 | 6.0 | -2.3 | 5.4 | -2.9 |
| Leu4 → *D*-CF_3_-Bpg | 9.2 | -4.1 | 9.3 | -4.0 | 9.4 | -4.1 |
| Leu5 → *D*-CF_3_-Bpg | 6.1 | -2.7 | 6.7 | -3.2 | 6.7 | -3.1 |
| Ile8 → *D*-CF_3_-Bpg | 6.4 | -3.0 | 6.4 | -3.1 | 6.5 | -3.7 |
| Leu10 → *D*-CF_3_-Bpg | 9.8 | -4.3 | 9.8 | -4.9 | 10.0 | -4.7 |
| Leu13 → *D*-CF_3_-Bpg | -7.5 | -7.5 | 6.0 | -3.0 | 6.1 | -2.8 |
| Leu16 → *D*-CF_3_-Bpg | -7.5 | -7.5 | -7.5 | -7.5 | -7.5 | -7.5 |
| Ile20 → *D*-CF_3_-Bpg | -7.5 | -7.5 | 8.3 | -3.9 | 8.3 | -3.7 |
| Leu21 → *D*-CF_3_-Bpg | -7.5 | -7.5 | 5.1 | -2.7 | 5.6 | -2.5 |

**Scheme S1: Chemical structure of *L*-CF_3_-Bpg (left) and *D*-CF_3_-Bpg (right).**


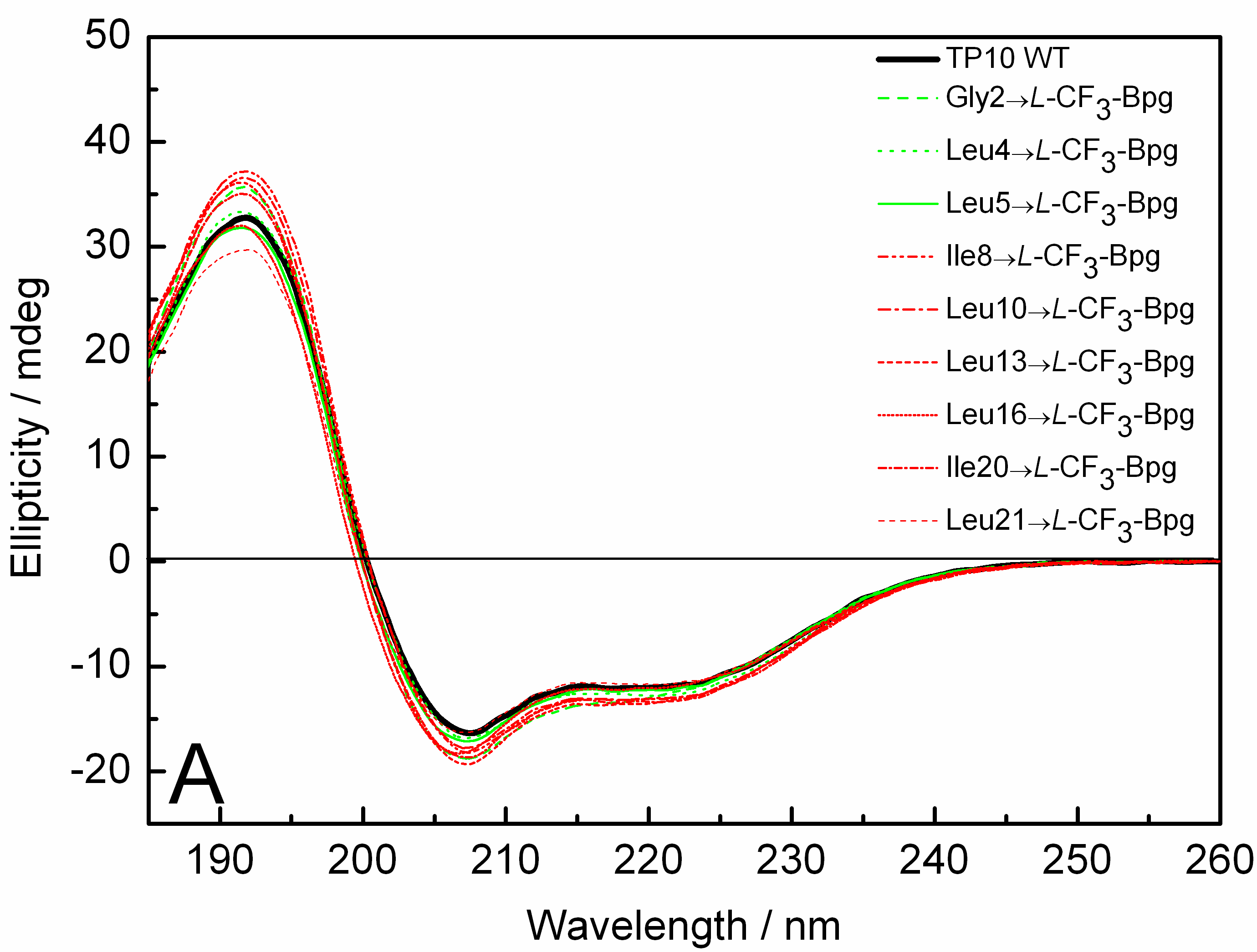

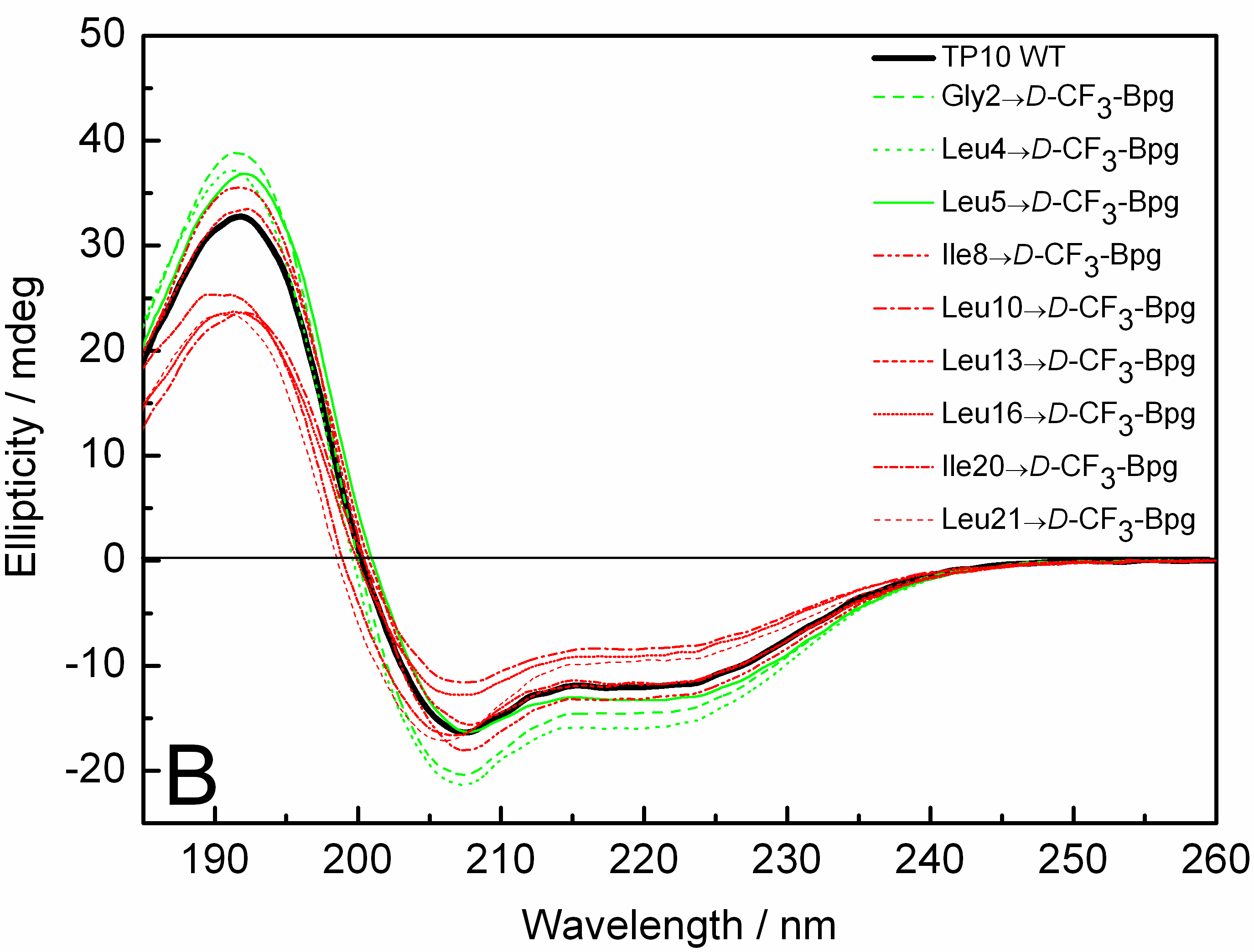


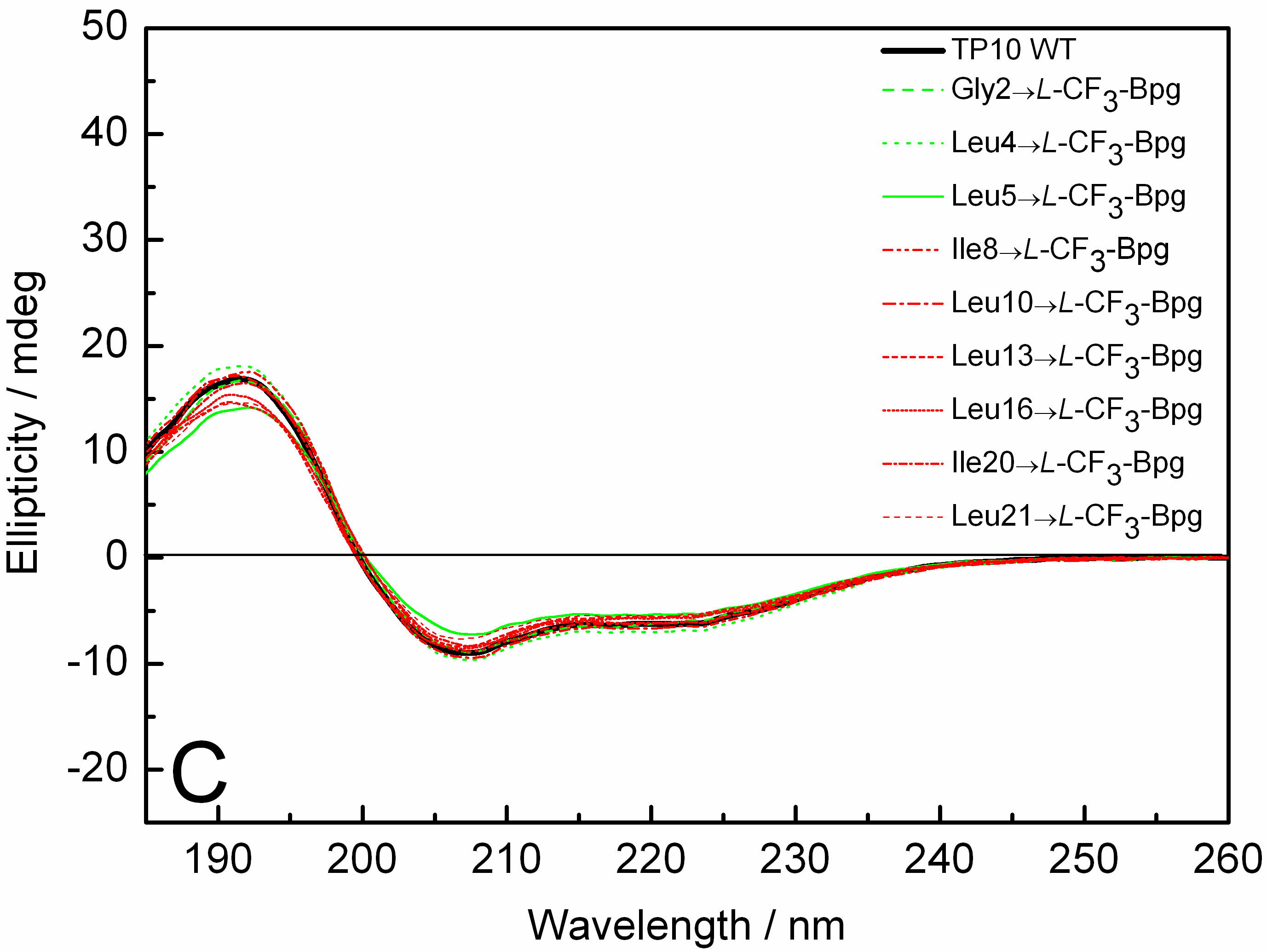

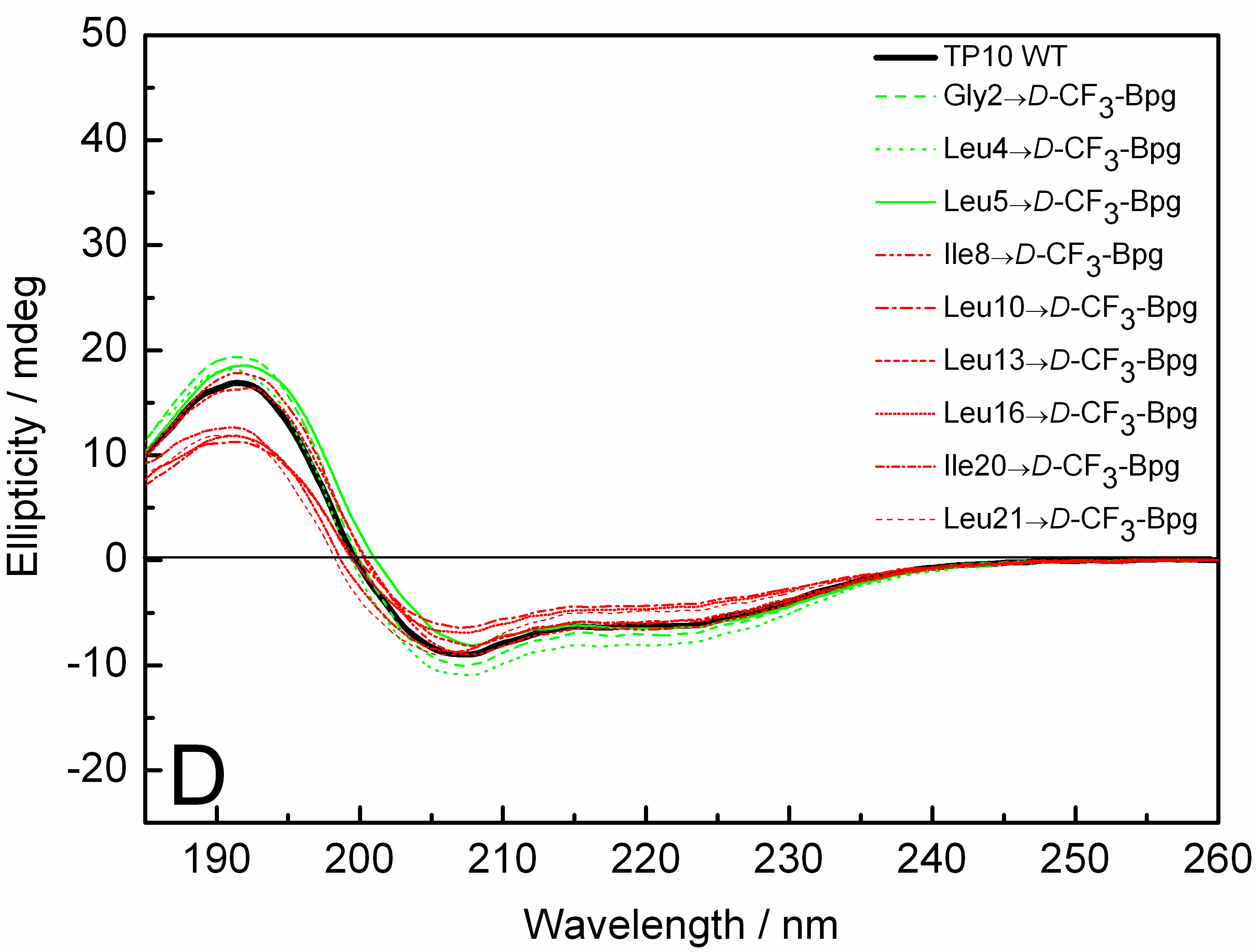


Figure S1: CD spectra of the CF_3_-Bpg labeled TP10 analogs bound to DMPC/DMPG vesicles. (A) *L*- and (B) *D*-epimers at P/L=1:50. (C) *L*- and (D) *D*-epimers at P/L=1:100. The wild type TP10 is shown as a black line; green lines show analogs with CF_3_-Bpg labels in the galanin part, and red lines in the mastoparan part.


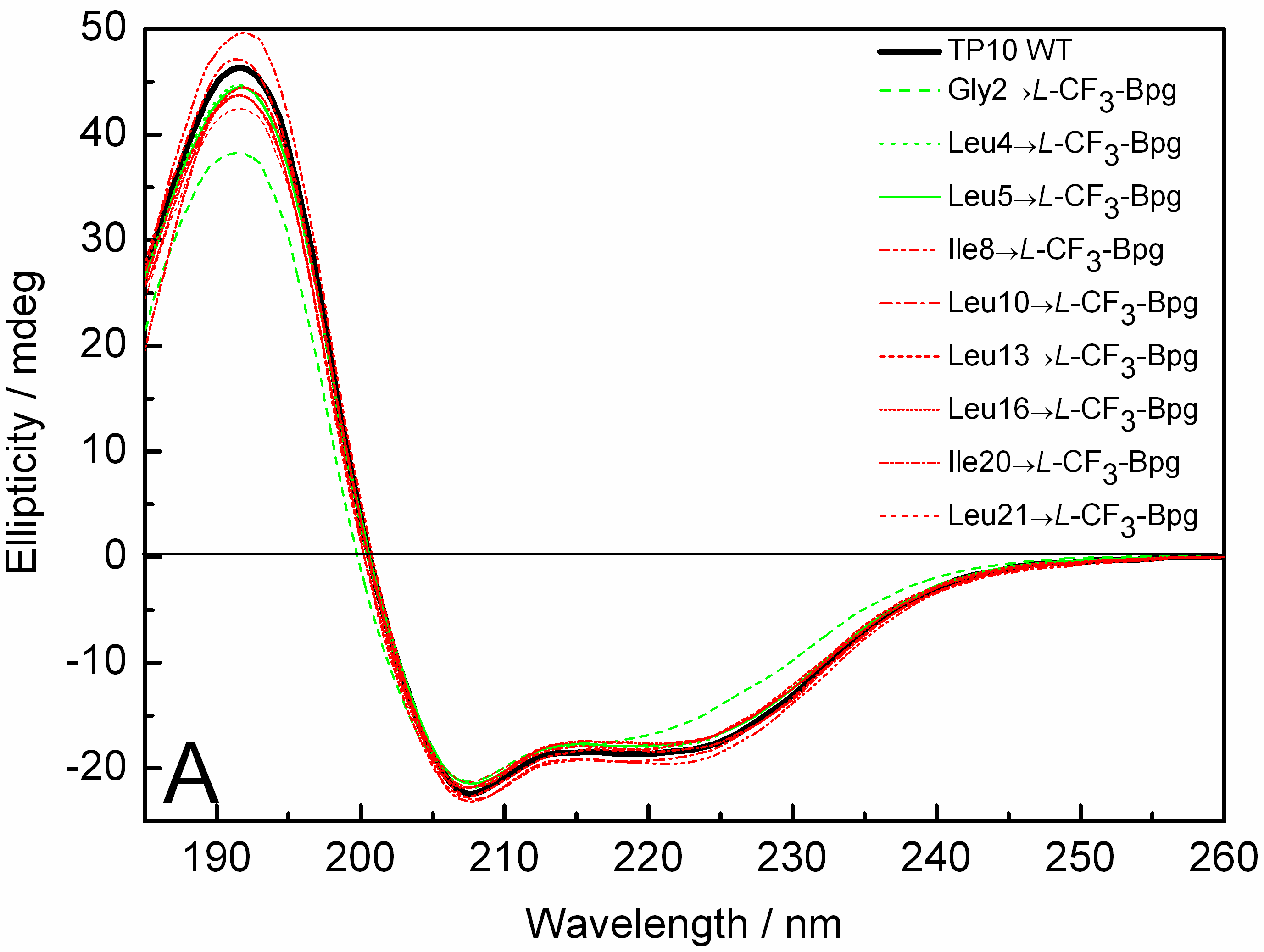

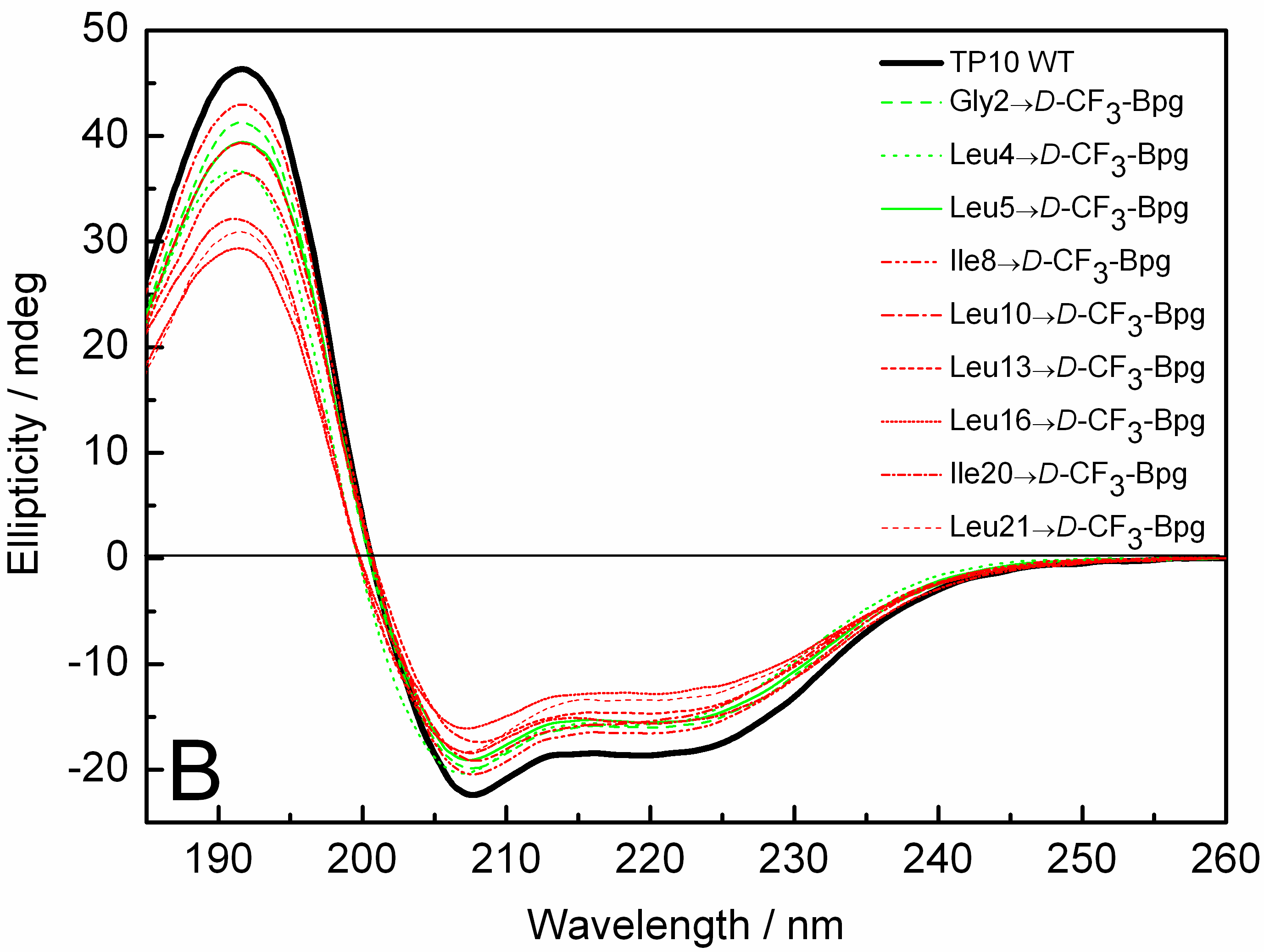
Figure S2: CD spectra of the carboxyfluorescein- and CF_3_-Bpg labeled TP10 analogs in DMPC/DMPG vesicles at P/L=1:50. (A) *L*- and (B) *D*-epimers. The wild type TP10 is shown as a black line; green lines show analogs with CF_3_-Bpg labels in the galanin part, and red lines in the mastoparan part.


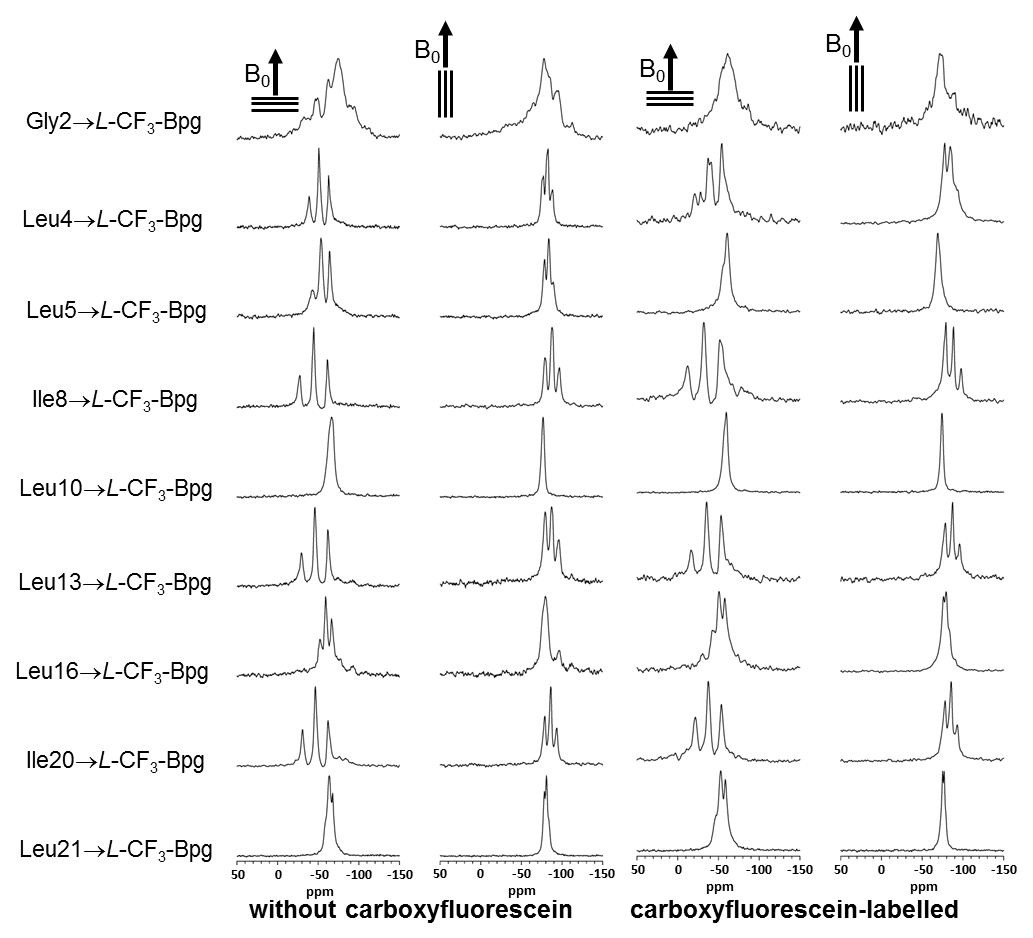


**Figure S3: Comparison of the solid-state ^19^F-NMR spectra of *L*-CF_3_-Bpg labeled TP10.** Analogs with and without the carboxyfluorescein-label were measured in oriented DMPC/DMPG bilayers at P/L=:200, at 40°C, with the sample normal aligned parallel (0°) and perpendicular (90°) to the static magnetic field**.**


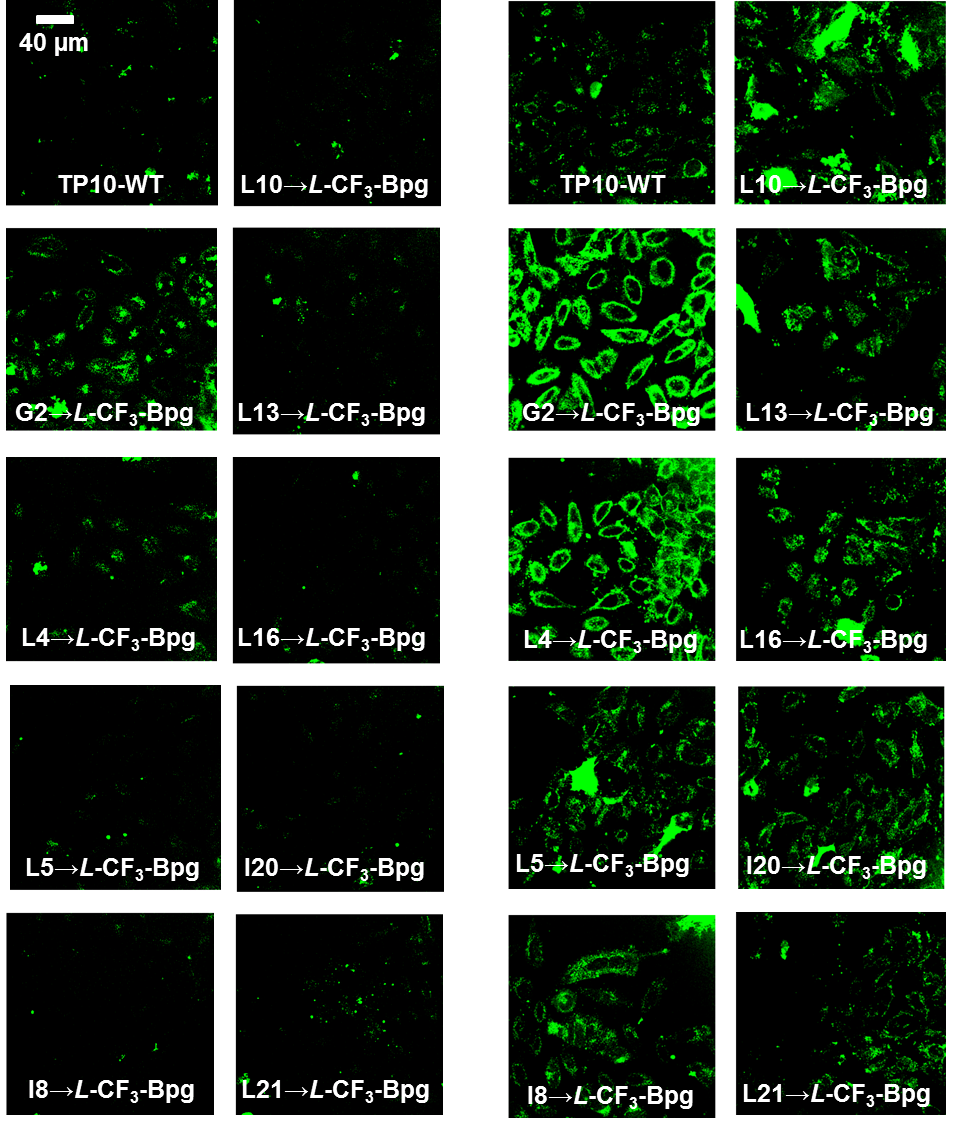


Figure S4: Internalization of the carboxyfluorescein-labeled TP10-WT and the *L*- and *D*-CF_3_-Bpg analogs by HeLa-cells. The cells were incubated with 2 µM (left column) and 10 µM (right column) peptide at 37°C for 30 min.

Figure S5: Solid-state ^19^F-NMR spectra of the *L*-CF_3_-Bpg labeled TP10. Analogs were measured in oriented DMPC/DMPG bilayers at P/L=1:50 and 1:200, at 40°C, with the sample normal aligned parallel (0°) and perpendicular (90°) to the static magnetic field B_0_. Several spectra showed an immobilized powder component, as indicated by the boxes.


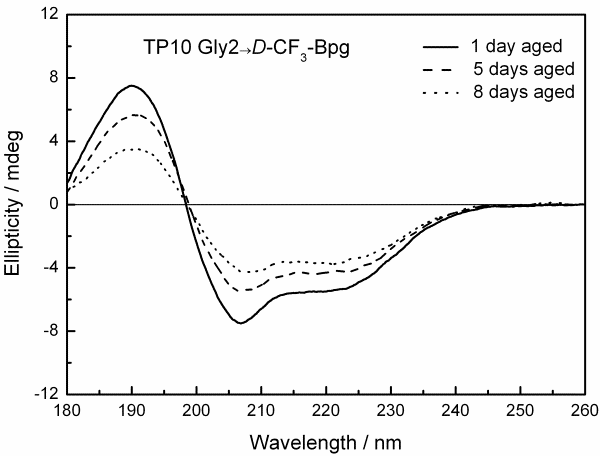

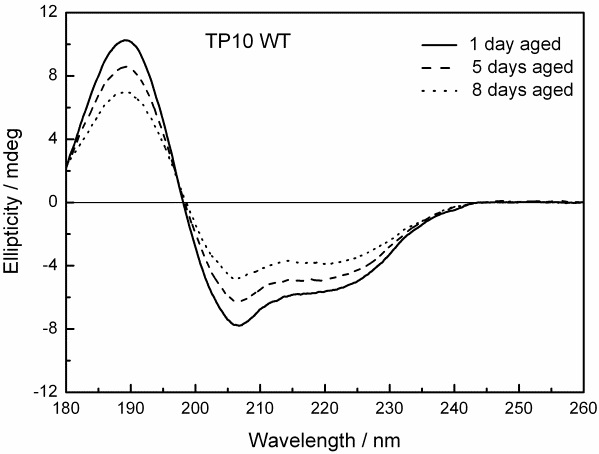

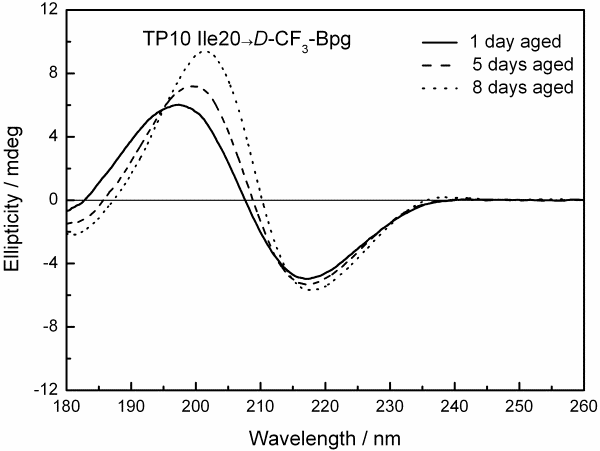

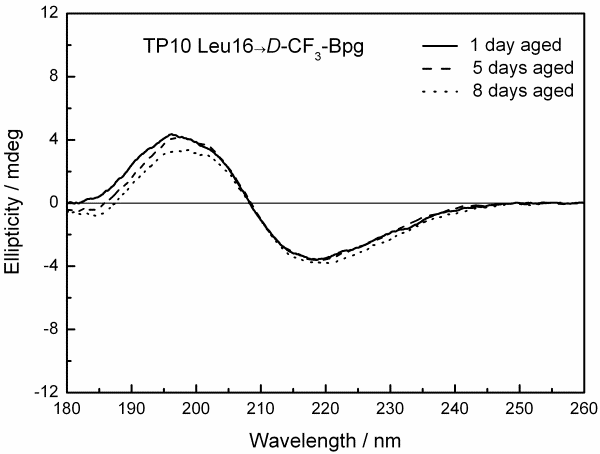

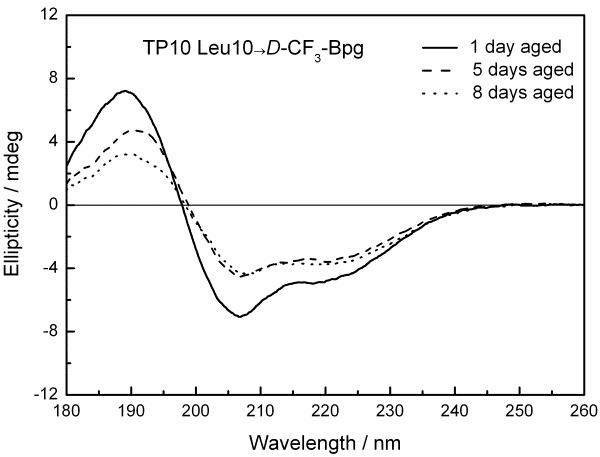

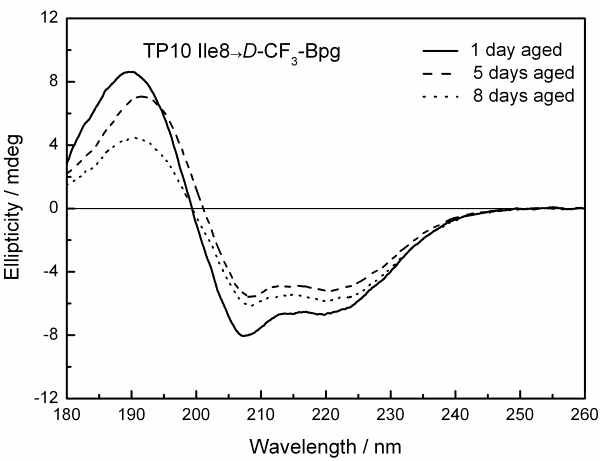

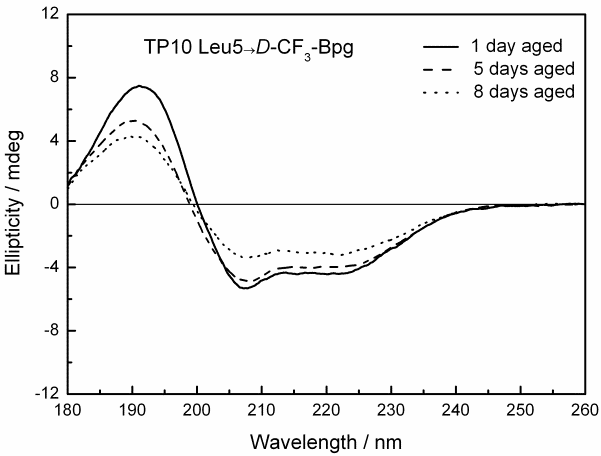

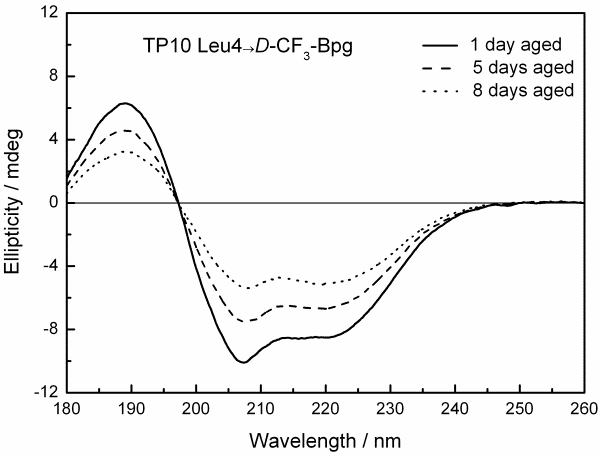


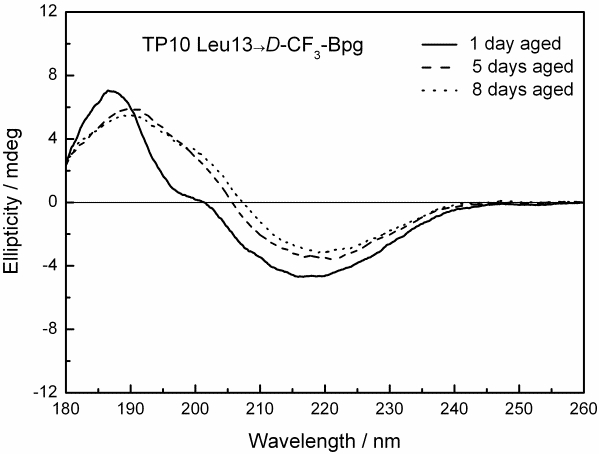


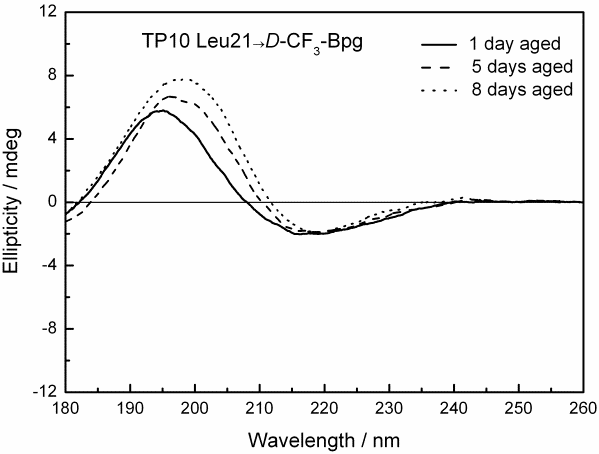


**Figure S6:** **OCD spectra of the *D*-CF_3_-Bpg labeled TP10**. Analogs were measured in oriented DMPC/DMPG bilayers at P/L=1:50. Spectra were recorded after 1 (straight lines), 5 (dashed lines) and 8 days (dotted lines) hydration of the OCD sample.

**
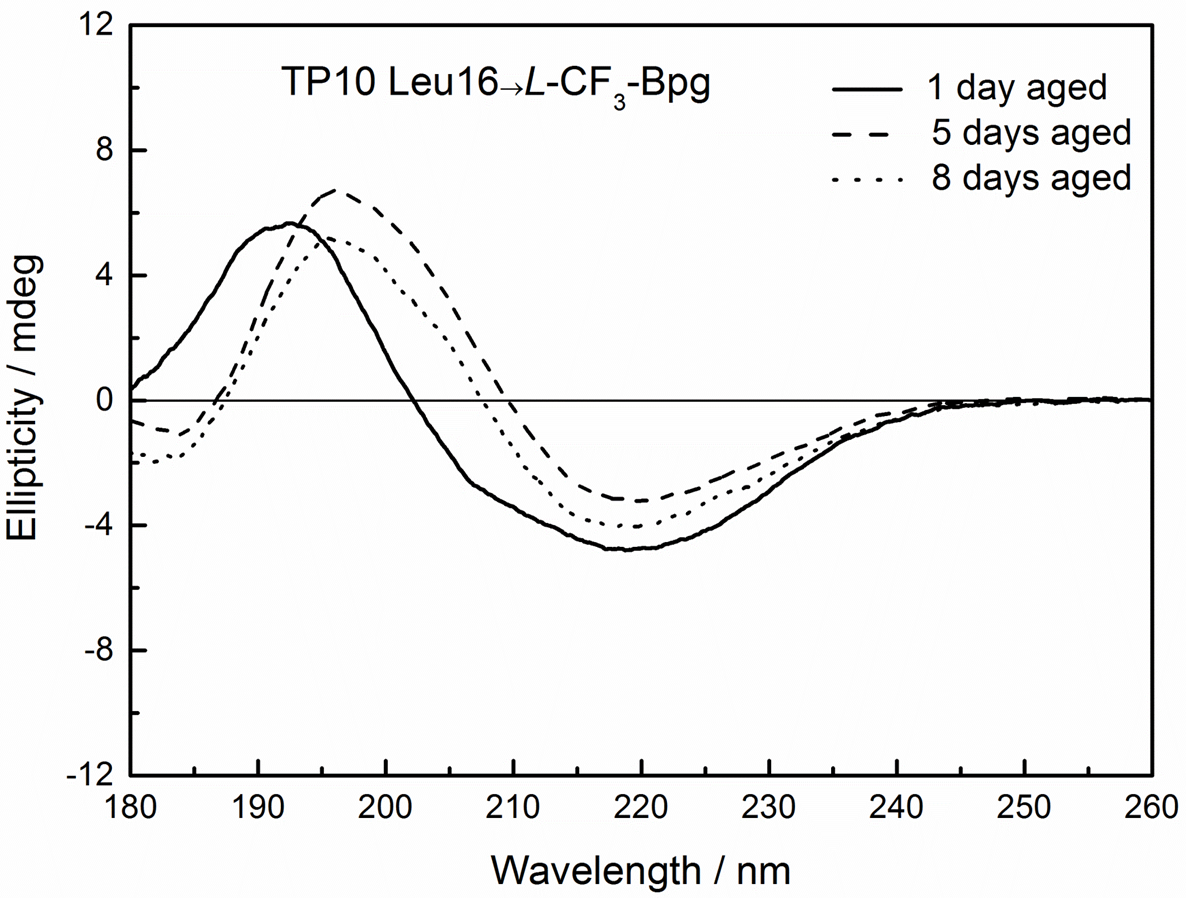
**

**Figure S7:** **OCD spectra of the Leu16→*L*-CF_3_-Bpg labeled TP10**. Peptide measured in oriented DMPC/DMPG bilayers at P/L=1:50. Spectra were recorded after 1 (straight lines), 5 (dashed lines) and 8 days (dotted lines) hydration of the OCD sample.
